# The Role of Atomistic Tip Morphology in Tip-Enhanced Electronic Energy Transfer

**DOI:** 10.1021/acs.nanolett.6c03248

**Published:** 2026-10-22

**Authors:** Pablo Grobas Illobre, Sveva Sodomaco, Piero Lafiosca, Tommaso Giovannini, Chiara Cappelli

**Affiliations:** † 226478 Scuola Normale Superiore (SNS), Piazza dei Cavalieri 7, 56126 Pisa, Italy; ‡ Department of Physics, 9318 University of Rome Tor Vergata, Via della Ricerca Scientifica 1, 00133 Rome, Italy

**Keywords:** Tip-enhanced, Fluorescence, FRET, Modeling, Multiscale, Atomistic

## Abstract

Tip-enhanced photoluminescence in scanning tunneling microscopy nanocavities has emerged as a powerful tool to probe intermolecular electronic energy transfer (EET) at the single-molecule level. Single-atom modifications at a metallic tip apex can drastically alter the near-field, yet their quantitative impact on intermolecular EET has remained elusive. By exploiting a multiscale fully atomistic quantum mechanical (QM)/electrodynamical approach, we show a nontrivial modulation of the EET-driven fluorescence, jointly determined by the atomistic morphology and single-atom features of the silver tip apex. Remarkably, we demonstrate that a fully atomistic QM/Classical description, able to treat systems of more than ten thousand atoms, is essential to capture how subtle apex rearrangements affect intermolecular EET. Our results quantitatively reproduce experimental fluorescence spectra and Förster-type distance behaviors, providing a direct structure–property connection at the atomistic level, crucial for predictive simulations of tip-enhanced spectroscopies.

## Graphical abstract

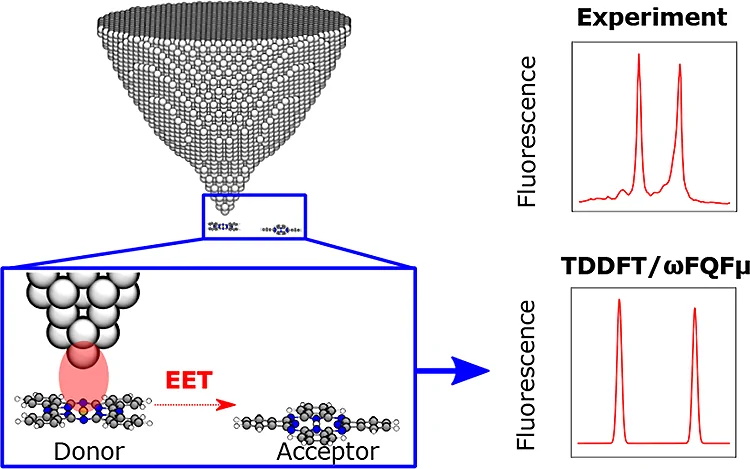

Plasmonic nanocavities formed by approaching a sharp metallic tip toward a metallic substrate can concentrate electromagnetic fields into subnanometric volumes, generating local enhancements that are at the basis of tip-enhanced spectroscopies.^1–3^ Tip-enhanced Raman scattering (TERS) and tip-enhanced photoluminescence (TEPL) have exploited such confinement to image single molecules with angstrom-scale spatial resolution, map vibrational fingerprints, and resolve submolecular electronic structure with high precision.^4–12^ The sensitivity of these techniques is directly governed by the local field distribution near the tip apex, which is ultimately determined by its atomistic morphology, especially considering that tip–substrate distances reach below 1 nm.^13–16^ Such a morphology dependence is pushed to its limit when picocavities are formed, i.e., plasmonic nanogaps featuring single-atom defects (adatoms).^17^ Such adatoms can induce drastic qualitative, and even quantitative, modifications to the optical environment and the spectral responses of adsorbed molecules within the nanogap.^10,17–23^ Determining how the tip morphology and its potential atomistic defects affect the molecular response thus represents a crucial step toward achieving reproducible, well-defined nanoscale optical measurements and for a proper theoretical rationalization.

In this work, we focus our attention on scanning tunneling microscopy (STM) nanocavities that provide an ideal platform for the real-space probing of intermolecular energy transfer. STM-induced luminescence (STML) and TEPL have been used to excite and detect individual chromophores adsorbed on ultrathin decoupling layers, resolving their photophysics at the single-molecule level, in terms of emission spectra, excited-state lifetimes, and electrofluorochromic switching, allowing for tip-assisted electronic energy transfer (EET) between neighboring molecular emitters.^8,24–32^ In all cases, the near-field enhancement is ultimately determined by the metallic tip morphology and determines the experimental measurement. However, the quantitative impact of the tip atomistic structure on intermolecular EET has remained largely unexplored.^33–35^ In fact, capturing this effect demands a fully atomistic description of both the metallic nanostructure and the molecular chromophores.

Here, we exploit a multiscale hybrid fully atomistic quantum-classical method to shed light on how the single-atom morphology of the scanning probe tip modifies the donor–acceptor EET between adsorbed chromophores.^36,37^ To focus on single-atom features, a fully atomistic picture of both the plasmonic nanostructure and the molecular systems is required. Thus, while the donor and acceptor chromophores are treated quantum mechanically (QM) by means of time-dependent density functional theory (TDDFT),^37^ we resort to the fully atomistic frequency-dependent fluctuating charges and fluctuating dipoles (ωFQFμ) model for treating the optical response of the metal nanoparticle.^38–51^ The ωFQFμ method is a purely classical approach which incorporates a physically consistent treatment of both intraband and interband transitions, achieving an accuracy comparable to that of first-principles methods, but at a much lower computational cost.^43,47,49^ The TDDFT/ωFQFμ approach thus retains a fully atomistic description of both the molecules and the tip, which allows single-atom features of the tip apex to be resolved, unlike other TDDFT/Classical approaches in which the tip is described as a continuum dielectric whose surface is discretized through the Boundary Element Method (BEM).^32,35,52^ The theoretical background of the TDDFT/ωFQFμ­(BEM) coupling (i.e., TDDFT coupled to an ωFQFμ or BEM tip or to a mixed ωFQFμ-BEM tip–base system^48^) is given in Section S2.

To analyze how variations in the tip structure modulate donor–acceptor EET efficiency, we use the experimentally studied systems in ref 52 as our reference. There, platinum phthalocyanine (PtPc) as the donor and zinc phthalocyanine (ZnPc) as the acceptor are adsorbed on a silver substrate, and the donor–acceptor EET is measured by imposing an external bias on the STM setup (Figure 1). In this work, such a bias is simulated by evaluating the TDDFT/ωFQFμ response at a fixed frequency ω, with the tunneling excitation applied only to the donor, and captured through electron withdrawal from the donor HOMO and injection into the degenerate LUMO or LUMO+1 with equal probability, following ref 35 (see Section S2.5 for further details). To reproduce the experimental geometry, we set the relative PtPc–ZnPc macrocycle rotation to 45° and the center-to-center distance to 2.21 nm, as shown in Figure 1. We consider four diverse Ag tips to study how they affect the calculated fluorescence emission. In particular, Tip-1 and Tip-2 (Figure 2b,c) model STM tips characterized by an atomically sharp apex, while Tip-3 and Tip-4 are paraboloid-like nanoparticles (see Figure 2d,e). Tip-2 and Tip-4 are obtained from Tip-1 and Tip-3, respectively, by removing the apex atom, thereby allowing us to quantify the effect of a single-atom defect at the top of the STM tip. All structures are composed of more than 14000 atoms (see Section S1 for further details). Note that the experimental reference includes an Ag metallic slab and a NaCl substrate on which the molecular systems are adsorbed. In our TDDFT/ωFQFμ modeling, the supporting slab is not explicitly included. However, within the experimental separation regime (15 Å gap between the molecule and the substrate mediated by NaCl buffer), we found that the substrate plays a negligible role in EET mechanisms (see Section 3.3).

**Figure 1:**
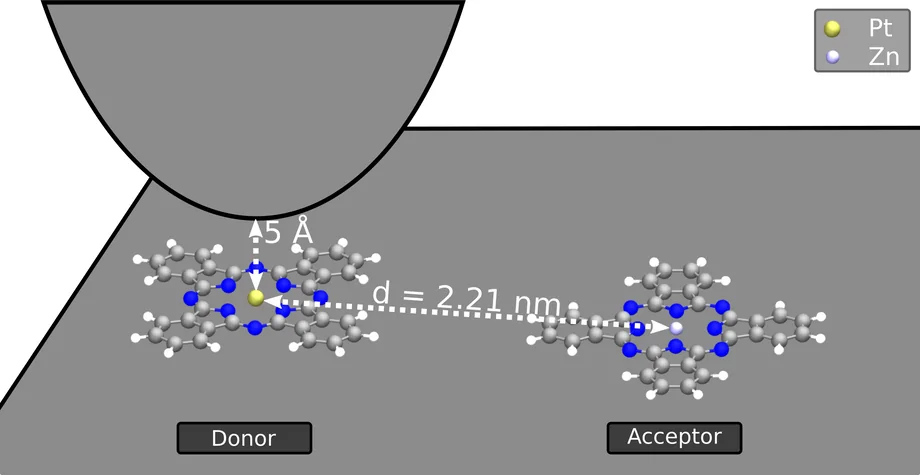
Schematic representation of the PtPc (donor)–ZnPc (acceptor) pair adsorbed on the Ag(111) substrate. The silver tip is placed 5 Å above PtPc (donor), and the intermetal Pt–Zn separation is kept fixed to 2.21 nm.

**Figure 2:**
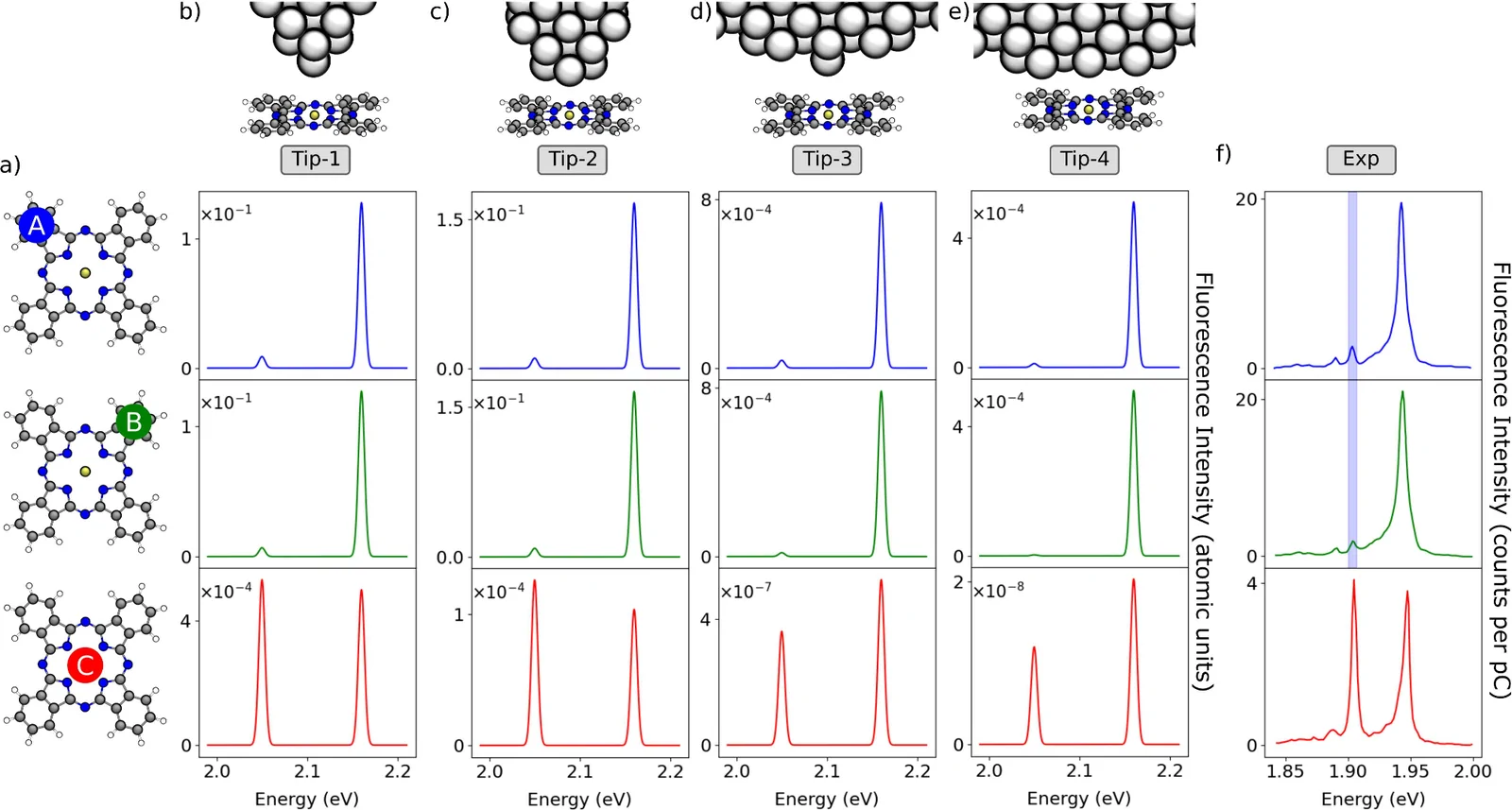
(a) Molecular geometry of PtPc, with the three tip positions A, B, and C highlighted. (b–e) Schematic depictions of Tip-1, Tip-2, Tip-3, and Tip-4 (top) and the corresponding absolute TDDFT/ωFQFμ fluorescence spectra at positions A (blue), B (green), and C (red) (bottom). (f) Experimental fluorescence spectra reproduced with permission from ref 52. Copyright 2022 Springer Nature. The shaded region highlights the PtPc emission band.

We first characterize the optical response of the four Ag tips (Figure S3) as calculated at the ωFQFμ level.^43,49^ All four structures exhibit a main absorption peak at about 3.2 eV, and a weaker shoulder at about 3.5 eV. Furthermore, Tip-1 and Tip-2 display an additional weaker shoulder at about 3.0 eV, originating from a *z*-polarized apex-edge plasmon mode sustained by the sharper tip geometry (Figure S4). Moreover, Figure S5 further shows the electric field enhancement maps, computed on the XY plane containing the molecules, for the four simulated atomistic tips. For comparison, we also consider four continuum smooth-tip models, investigated at the BEM level of theory.^53,54^ Continuum Tip-1 and Continuum Tip-2 reproduce the surface of the atomistic Tip-1 and Tip-2, respectively, the apex atom of Tip-1 being mimicked by a half-embedded sphere, whereas Continuum Tip-α and Continuum Tip-β have geometries analogous to those used in refs 35 and 52, respectively (Figure S6). By using the Brendel–Bormann permittivity, Continuum Tip-1 shows a main broad peak at 2.50–2.55 eV and a secondary peak at 3.36 eV, while Continuum Tip-2 presents a main peak centered at 2.55 eV and two higher energy signals at 3.0 and 3.37 eV. Furthermore, Continuum Tip-α shows a main peak at 3.0 eV and two shoulders at 3.4 and 3.7 eV, while Continuum Tip-β exhibits three signals at 2.7, 3.1, and 3.6 eV. More in detail, all Continuum Tip-1 signals display a mixed *x*, *y*, and *z*-polarization character, with a weaker contribution of the *z*-polarized plasmonic mode (i.e., along the tip’s principal axis), whereas for Continuum Tip-2 only the signals at 3.0 and 3.37 eV show a *z*-polarized character. For Continuum Tip-α, the absorption peaks dominated by a *z*-polarized mode are those at 3.0 and 3.35 eV, while for Continuum Tip-β this is the case only for the signal at 2.7 eV. Notably, Continuum Tip-α shows the best qualitative agreement with the atomistic ωFQFμ absorption spectra (Figures S3 and S6).

We next focus on tip-mediated intermolecular EET, using the experimental work as a reference.^52^ For all tip morphologies, the intermolecular EET rate from donor state *i* to acceptor state *j* is calculated as^35,55^

1 $$\Gamma_{\mathrm{EET}}^{i\to j}=\frac{2\pi }{\hbar }|V_{ij}{|}^{2}J$$

where *V*_*ij*_ represents the electronic coupling between the donor and acceptor states, including the polarization effects induced by the silver tip, ℏ is the reduced Planck constant, and *J* denotes the spectral overlap integral derived from the experimental donor emission and acceptor absorption spectra (see also Section S2.4).^29,52^ The fluorescence signals associated with the donor (*I*_D_) and acceptor (*I*_A_) molecules can then be calculated as follows:^35^

2a $$I_{D}^{i}=\Gamma_{\mathrm{ex},D}^{i}\Phi_{D}^{i}$$

2b $$I_{A}^{i}=\Gamma_{\mathrm{ex},D}^{i}\underset{j}{\sum }\eta_{\mathrm{EET}}^{i\to j}\Phi_{A}^{j}$$

where Γ_ex,*X*_^*i*^ and Φ_*X*_^*i*^ are the excitation rate and fluorescence quantum yield associated with state *i*, respectively, and *X* ∈ {D, A} identifies either the donor or acceptor species. In eq 2b, η_EET_^*i*→*j*^ corresponds to the EET efficiency from donor state *i* to acceptor state *j*. More specifically, η_EET_^*i*→*j*^ represents the probability of energy transfer, defined as the fraction of the donor state *i*’s total decay rate that proceeds via EET exciting the acceptor into state *j* (see Section S2.5 for more details).

In our simulations, the tips are placed 5 Å above PtPc, measured from the closest atom, at three molecular sites denoted as A, B, and C (Figure 2a). All TDDFT/ωFQFμ simulations are performed at the fixed frequency ω = 2.5 eV (Figure 2b–e; experimental bias −2.8 V; see Section S2.5 for the tunneling excitation scheme). The experimental fluorescence spectra are also graphically depicted in Figure 2f.

All QM/ωFQFμ fluorescence spectra are characterized by two main signals: a lower-energy ZnPc emission at 2.05 eV and a higher-energy PtPc emission at 2.16 eV. Experimentally, the two bands are reported at 1.898–1.907 eV for ZnPc and 1.940–1.949 eV for PtPc. Clear trends emerge as a function of the tip position. In particular, when positions A and B are considered, in all simulated spectra, the lower-energy signal is much weaker than the higher-energy one, correctly reproducing the experimental measurements. In contrast, at site C, the fluorescence spectra significantly depend on the tip morphology. When the tip features an atomically sharp apex (Tip-1 and Tip-2; Figure 2b,c, bottom), the two signals become comparable in intensity, with a slight predominance of the lower-energy peak. The opposite trend is instead reported for Tip-3 and Tip-4 (Figure 2d,e, bottom). In all cases, an overall reduction in absolute emission is reported. Remarkably, we note that the presence of a single atom defect does not significantly alter the relative intensities of the two bands, but it does have an impact on the absolute intensity, with an increase of an order of magnitude for both Tip-1 and Tip-3. By comparing our results with the experiments, we note that Tip-1 and Tip-2 TDDFT/ωFQFμ calculations reproduce the experimental reference almost perfectly, capturing the relative peak positions, the intensity pattern, and the qualitative quenching observed at site C. Only a slight discrepancy in the absolute intensity at position C is reported, which can be associated with the ideal modeling performed in our simulations that do not consider vibronic effects.^56^ In particular, moving from Tip-1 to Tip-2, removing the apex atom slightly increases the absolute TDDFT/ωFQFμ fluorescence intensity at positions A and B, while decreasing it at position C (5.32 × 10^–4^ → 1.26 × 10^–4^). Instead, moving from Tip-3 to Tip-4, removing the apex atom decreases the absolute intensity at all three positions, being sharply quenched at position B by about 1 order of magnitude (1.85 × 10^–5^ → 3.26 × 10^–6^; Table S1). Finally, we analyze the dependence of the simulated spectra on the atomistic vs continuum description of the tip (Table S1 and Figure S7) and on the selected simulation frequency (Figure S8).

To quantify these trends, Figure 3 gathers the resonance energy transfer efficiency, RET_eff_ = *I*_A_/(*I*_D_ + *I*_A_),^35^ at positions A, B, and C. Here, Tip-1 (A, 0.07; B, 0.05; C, 0.52) and Tip-2 (A, 0.07; B, 0.045; C, 0.55) give similar values, as do Tip-3 (A, 0.05; B, 0.02; C, 0.41) and Tip-4 (A, 0.03; B, 0.005; C, 0.37). The results obtained at the TDDFT/BEM level show instead marked differences among the four tips considered: Continuum Tip-α signals (A, 0.08; B, 0.035; C, 0.77) vary substantially across the three positions, while Continuum Tip-1, Continuum Tip-2, and Continuum Tip-β results remain close to zero throughout. Experimentally, RET_eff_ is 0.12, 0.08, and 0.52 at positions A, B, and C, respectively.

**Figure 3:**
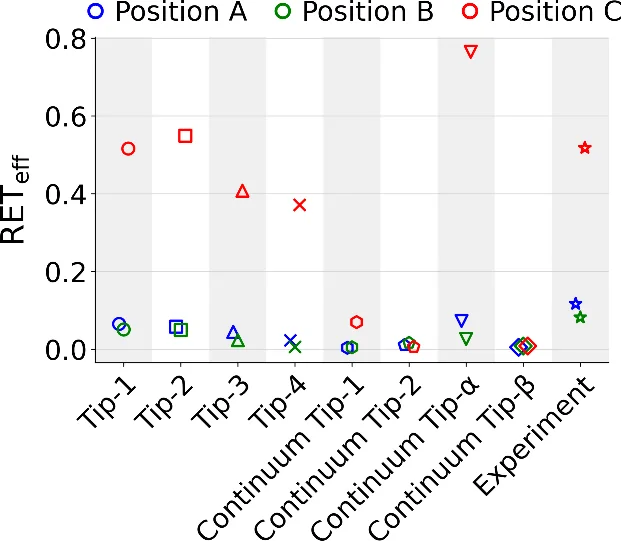
RET efficiency simulated at tip positions A (blue), B (green), and C (red), for the four atomistic tip models, the four continuum tip models, and the experimental reference. Adapted with permission from ref 52. Copyright 2022 Springer Nature.

TDDFT/ωFQFμ RET results simulated with Tip-1 and Tip-2 best reproduce the experimental trend across all three positions. Notably, RET_eff_ at position C increases from Tip-1 to Tip-2 but decreases from Tip-3 to Tip-4 upon apex-atom removal, suggesting that this behavior arises from a nontrivial competition between the electronic energy transfer rate and the radiative and nonradiative decay pathways of both donor and acceptor, governed jointly by the overall tip morphology and by single-atom details (see Figure S11 for the individual rates). At every position, TDDFT/ωFQFμ results simulated with Tip-1 and Tip-2 give a higher RET_eff_ than those simulated with Tip-3 and Tip-4, suggesting that the overall tip morphology sets the dominant baseline for the EET response, with the single-atom apex defect acting as a secondary, morphology-dependent modulation. Conversely, TDDFT/BEM results simulated with Continuum Tip-α overestimates RET_eff_ sharply at C, while Continuum Tip-1, Continuum Tip-2, and Continuum Tip-β results miss the trend at all three positions. Thus, our results suggest that an implicit (nonatomistic) modeling of the tip is insufficient to reproduce the experimental tip-mediated EET response, requiring a fully atomistic QM/Classical simulation to effectively capture the underlying physics that drives the total fluorescence signal. In fact, tip-related quantities alone, such as the electric field enhancement, cannot predict our TDDFT/ωFQFμ results (Figure S5).

Given the pronounced sensitivity of the fluorescence signal when the tip is positioned above the Pt atom (position C), we next examine the distance dependence of intermolecular EET while keeping the tip fixed at site C. In Figure 4, we report the calculated PtPc and ZnPc fluorescence intensities as a function of the donor–acceptor intermetal distance and tip morphologies as calculated at the TDDFT/ωFQFμ level (Figure 4a–d). In all simulations, the PtPc and the tip are kept fixed, while ZnPc is translated longitudinally. The simulated intensities are normalized to the experimental ZnPc signal at the shortest separation considered (*d* = 1.72 nm). For direct comparison, we also include the Förster resonance energy transfer (FRET) fit to the experimental ZnPc intensity, using the functional form *I*_ZnPc_(*d*) = 1/(*A* + *Bd* ^6^), where *A* and *B* are fitting parameters (see also Section S2.6).^52^

**Figure 4:**
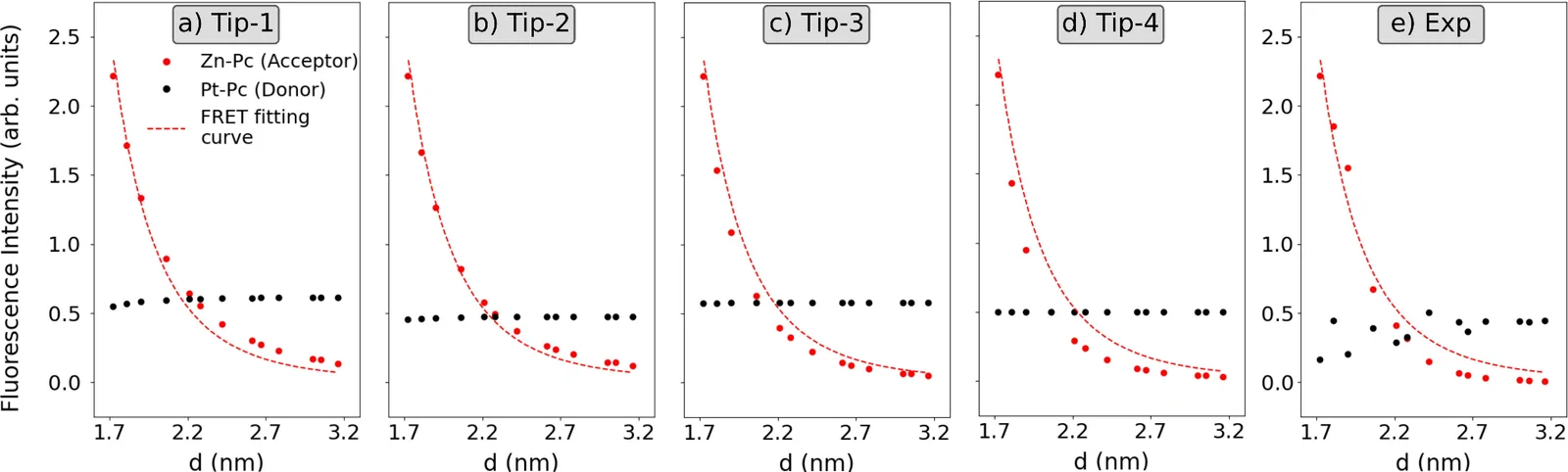
TDDFT/ωFQFμ PtPc and ZnPc fluorescence intensities as a function of the donor–acceptor intermetal distance for (a) Tip-1, (b) Tip-2, (c) Tip-3, and (d) Tip-4 morphologies with the tip positioned at site C. The simulated curves are normalized to the experimental ZnPc intensity at (*d* = 1.72) nm. Experimental data are reproduced in panel e with permission from ref 52. Copyright 2022 Springer Nature.

In all simulated tip geometries, the ZnPc fluorescence exponentially decreases with distance, consistent with the characteristic FRET-like *d* ^–6^ behavior. This is in good agreement with the experimental reference (Figure 4e). This trend supports the exclusion of Dexter-type contributions in our simulations, which are expected to be negligible at the donor–acceptor separations considered, as also corroborated by the agreement with the experiment.^29,35,52^ Remarkably, the atomistic features of the tip also affect the EET as a function of the distance. Indeed, the Tip-1 simulations show a slight but monotonic increase in PtPc fluorescence by increasing the intermolecular distance (from 0.55 to 0.61 au between *d* = 1.72 and 3.16 nm). For the other tip geometries, the PtPc fluorescence remains comparatively flat, whereas the ZnPc signal retains its FRET-like *d* ^–6^ decay. Notably, the ZnPc decay becomes progressively steeper from Tip-2 to Tip-3 and Tip-4. Furthermore, for Tip-3 and Tip-4, the ZnPc intensity falls below the experimental FRET fit, leading at *d* = 2.21 nm to an inversion of the relative peak intensities compared with Tip-1 and Tip-2. Our results demonstrate once again the delicate relationship between the tip morphology and the FRET decay profile and, consequently, the overall fluorescence response of the donor–acceptor system, with atomistic defects playing a relevant role in determining the molecular response

Figure 5 further examines the atomistic tip description’s influence on the EET process by reporting the corresponding RET efficiency as a function of donor–acceptor distance for the four atomistic tips, the four continuum tips, and the experiment. At short distances (*d* < 2.4 nm), TDDFT/ωFQFμ results simulated with Tip-1 and Tip-2 closely reproduce the experimental trend, whereas at larger distances (*d* ≥ 2.4 nm) the simulated results using Tip-3 and Tip-4 provide a better match. In contrast, TDDFT/BEM results simulated with Continuum Tip-α provide a good reproduction of the experimental value only at the shortest distance considered, with RET_eff_ strongly overestimated as the donor–acceptor distance increases, while Continuum Tip-1, Continuum Tip-2, and Continuum Tip-β underestimate RET_eff_ at all distances. These results highlight again that a continuum description cannot properly model tip-mediated EET response, even when resembling its atomistic counterpart. The corresponding fluorescence spectra mediated by the four continuum tips as a function of distance, as well as a study of the effect of the simulation frequency, are reported in Figures S9 and S10 of the SI, respectively.

**Figure 5:**
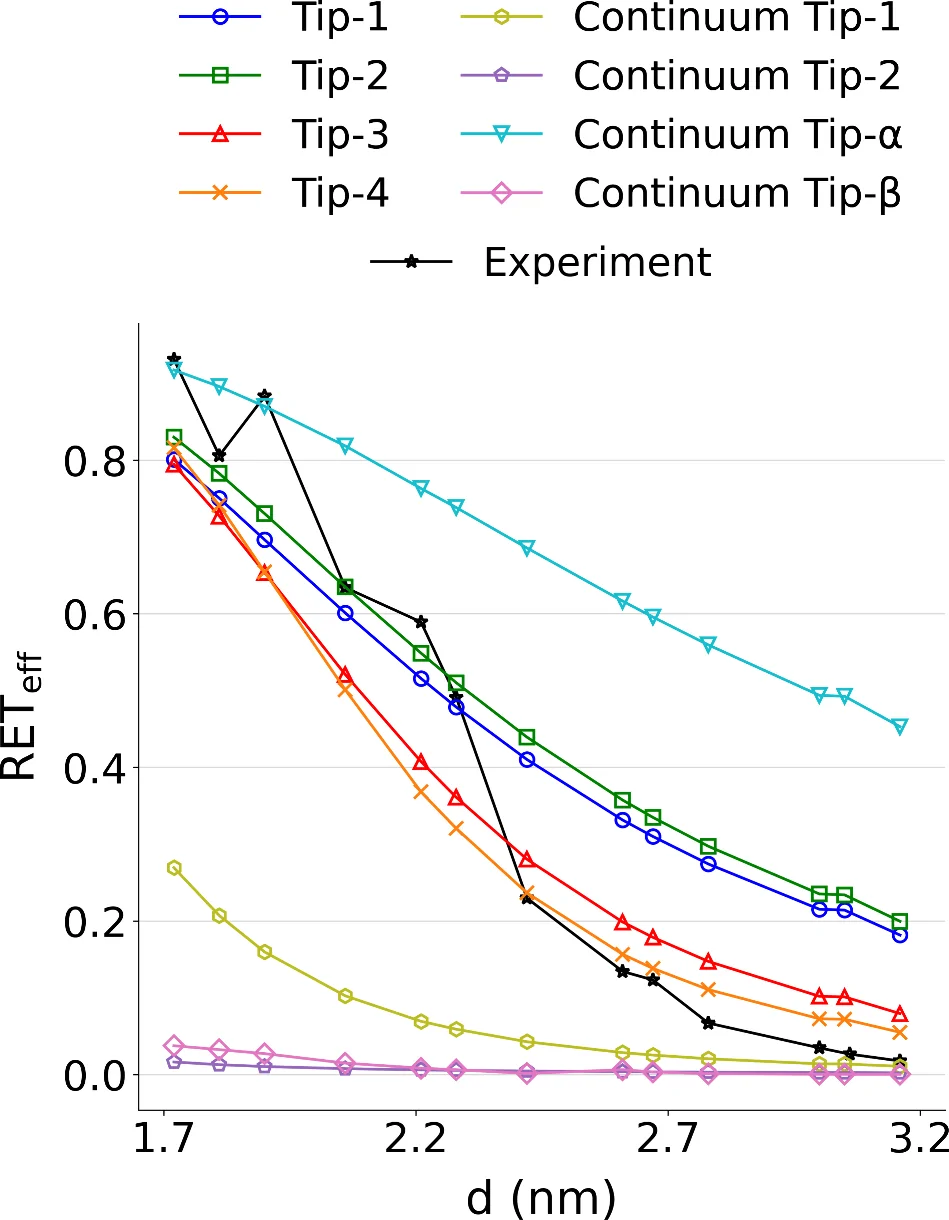
RET efficiency as a function of the donor–acceptor intermetal distance, with the tip positioned at site C, for the four atomistic tip models, the four continuum tip models, and the experimental reference. Adapted with permission from ref 52. Copyright 2022 Springer Nature.

Understanding the observed RET_eff_ trends requires analyzing how the quantities entering eqs 2a and 2b vary with distance. To this end, the donor excitation rate, the donor–acceptor EET rate, and the radiative and nonradiative decay rates of both molecules are reported in Figures S11 and S12 for TDDFT/ωFQFμ and TDDFT/BEM simulations, respectively. Specifically, moving from Tip-1 to Tip-2, the donor excitation rate remains nearly constant while its nonradiative (radiative) decay rate increases (decreases), and both acceptor decay rates slightly increase; together these changes modestly raise the EET rate and, with it, RET_eff_ at all distances for Tip-2. Moving from Tip-3 to Tip-4, instead, the donor excitation rate decreases, its nonradiative (radiative) decay rate increases (decreases), while the acceptor’s decay rates stay similar to distance; this yields a slightly larger EET rate, and hence RET_eff_, for Tip-3. In contrast, Continuum Tip-α combines uniquely low donor nonradiative and radiative decay rates with an acceptor radiative decay rate that strongly increases with distance, raising the fluorescence emission probability; together with a smoothly decreasing EET rate, this explains its high RET_eff_ values at increasing distance. Finally, the lower RET_eff_ values obtained for Continuum Tip-1, Continuum Tip-2, and Continuum Tip-β stem from their markedly larger donor radiative decay rates, which favor donor emission over EET.

To conclude, our work demonstrates the importance of an atomistic description of the tip apex when simulating donor–acceptor EET. By computing fluorescence emission from a donor–acceptor pair under the effect of different tip geometries, we demonstrate the intricate modulation of the EET process, driven by both the modifications in the overall tip morphology and the single-atom features at the tip apex. These results highlight the need for precise control of the tip’s atomistic profile in experimental setups, and its importance for achieving reproducible, well-controlled tip-enhanced measurements.^8,11,29,30,52,57^

Furthermore, our TDDFT/ωFQFμ protocol for EET advances the state-of-the-art modeling of tip-enhanced setups by offering a fully atomistic approach with accuracy comparable to full *ab initio* methods, allowing for the simulations of large and atomistically defined nanostructures. In this way, we open the way to predictive *in silico* design of tip-enhanced experiments and provide practical guidelines for achieving reproducible, optimized conditions in surface-enhanced applications such as molecular sensing and imaging.^1,3,58–62^ Finally, our multiscale TDDFT/ωFQFμ approach does not capture quantum effects requiring explicit overlap between the molecular and metallic states, such as charge transfer or orbital hybridization, expected to become relevant only at chemisorption-range separations shorter than those considered here.

## Supplementary Material



## Notes

Computational details, TDDFT/Classical model, spectral overlap calculation, FRET fitting curve parameters, absorption spectra of tips, electric field enhancement maps, TDDFT/Classical tip simulations, and ωFQFμ-BEM Tip-1–base (PDF)
