# Supplementary material for: The Role of Atomistic Tip Morphology in Tip-Enhanced Electronic Energy Transfer
Source: Nano Lett. 2026 Oct 22;26(43):14907–13. doi: 10.1021/acs.nanolett.6c03248 (PMC13741917; doi:10.1021/acs.nanolett.6c03248)
Supplement: Supplementary file 1 [file nl6c03248_si_001.pdf]

Supporting Information for: The Role of Atomistic Tip Morphology in Tip-Enhanced Electronic Energy Transfer

Pablo Grobas Illobre,[†] Sveva Sodomaco,[†] Piero Lafiosca,[†] Tommaso Giovannini,[*,‡] and Chiara Cappelli[*,†]

†*Scuola Normale Superiore, Piazza dei Cavalieri 7, 56126 Pisa, Italy.*

‡*Department of Physics, University of Rome Tor Vergata, Via della Ricerca Scientifica 1, 00133 Rome, Italy*

E-mail: tommaso.giovannini@uniroma2.it; chiara.cappelli@sns.it

S1 Computation Details

All ωFQFμ calculations were carried out with the `plasmonX` code,[1] whereas ωFQFμ-BEM calculations were performed with a development version of the program.[2] TDDFT/ωFQFμ simulations were performed with a locally modified version of the Amsterdam Modeling Suite (AMS) package,[3,4] and Eq. (S9) was evaluated in post-processing from the TDDFT/ωFQFμ outputs using an external parallelized Fortran code named FretLab (commit: `ff59168`).[5]

PtPc and ZnPc were used as donor and acceptor molecules, respectively. The initial molecular geometries were taken from Ref. 6, replacing the central metal as required, and were then studied at the *ab initio* level at the CAM-B3LYP/DZP level. The structures were first optimized *in vacuo*, and their optical responses were computed at the TDDFT level, including the polarization induced by the silver tip, using an external excitation frequency of $\omega = 2.5$ eV for the ωFQFμ calculations and of $\omega = 2.05$ eV for ωFQFμ-BEM studies. In all tip-only simulations, the molecules were placed on the XY plane, while the main axis of the tip was aligned along Z. The tip apex was positioned 5 Å above PtPc, measured relative to the closest molecular atom. In the tip–base simulations, the same tip–molecule arrangement was retained, while the base was placed 15 Å below the molecular plane, with the distance again measured relative to the closest molecular atom.

Silver tips were generated with the GEOM v1.0.0 Python package (commit: `72bb6994`).[7] Tip-1 (14735 atoms) and Tip-2 (14734 atoms) were built using the `microscope` option with `z_max_paraboloid`=60.0, `a`=0.023, `b`=0.023, `z_max_pyramid`=40.0, and `base_side_length`=40.0; Tip-2 was obtained from Tip-1 by removing the apex atom. Tip-3 (17059 atoms) and Tip-4 (17058 atoms) were generated with the `paraboloid` option using `z_max`=75.0, `a`=0.03, and `b`=0.03; Tip-4 was obtained from Tip-3 by removing the apex atom. In all atomistic tip models, the upper-section top-base diameter was approximately 95 Å, the distance from the top-base center to the apex was approximately 75 Å, and the silver lattice constant was set to 4.09 Å. The BEM cube (side length = 90 Å) employed in the ωFQFμ-BEM calculations, as well as the continuum smooth-tip geometries employed in the pure BEM simulations, were generated with the Gmsh package.[8 8] Continuum Tip-1 and Continuum Tip-2 are analogous to the atomistic Tip-1 and Tip-2, whereas Continuum Tip-α is analogous to the tip used in Ref. 6 and Continuum Tip-β to that used in Ref. 9. Continuum Tip-1 and Continuum Tip-2 were built as the union of a paraboloid and a square-based pyramid

reproducing the surface of the atomistic Tip-1. In Continuum Tip-1, a half-embedded sphere (radius = 1.8 Å) at the apex, smoothly blended into the pyramid faces, mimics the apex atom; in Continuum Tip-2, the apex instead ends in a flat facet with rounded edges.

For the ωFQFμ calculations, the silver parameters were taken from our previous works.[1,10] In the ωFQFμ-BEM setup, the BEM response was described using the dielectric function of Etchegoin *et al.*[11] fitted by Johnson and Christy[12,13] for silver. For the pure BEM continuum tip calculations, the optical response of Continuum Tip-1, Continuum Tip-2, Continuum Tip-α and Continuum Tip-β was evaluated using the Dipolar Polarizable Continuum Model (DPCM), combined with three silver permittivity functions: Brendel-Bormann,[14] Johnson and Christy,[15] and Palik.[16] For the tip-mediated fluorescence calculations, the Brendel-Bormann permittivity was selected for all continuum-tip simulations. The spectral overlap ($J = 2.968 \times 10^{-2}$ eV^{-1}) was computed from the experimental spectra reproduced in Fig. S1 from Ref. 9. To account for the double degeneracy of the molecules, EET was evaluated between the two lowest-energy donor and acceptor states, i.e., the first bright transitions. Consistent with previous modeling of related systems, the same J value was assigned to each donor–acceptor state pair.[6] Finally, $\Gamma_{\mathrm{nr},0}$ was obtained by first computing the vacuum radiative decay rate $\Gamma_{\mathrm{rad},0}$ at the TDDFT level, and then combining it with the experimental fluorescence quantum yield of the corresponding metal phthalocyanine, via $\Phi_0 = \Gamma_{\mathrm{rad},0}/(\Gamma_{\mathrm{rad},0} + \Gamma_{\mathrm{nr},0})$. We used $\Phi_{0,\mathrm{ZnPc}} = 0.3$ for the acceptor, and $\Phi_{0,\mathrm{PtPc}} \approx \Phi_{0,\mathrm{PdPc}} = 5.0 \times 10^{-4}$ for the donor, approximated by the measured quantum yield of PdPc.[17,18]

S2 Theory

S2.1 The ωFQFμ model

ωFQFμ represents the state-of-the-art for studying nanoplasmonics from a full atomistic, yet classical, perspective.[1,10,19–27] ωFQFμ combines a Drude-like conduction formalism with the Fluctuating Charges approach and quantum corrections to accurately model charge redistribution between atoms. In this way, through the computation of frequency-dependent atomic charges ($\mathbf{q}$), the model captures intraband contributions to the optical response.[19] The approach extends the description through the computation of fluctuating atomic dipoles (μ) to account for interband effects, utilizing a frequency-dependent atomic polarizability derived from experimental permittivity data.[10,25]

For a system formed by N atoms, the ωFQFμ linear equation reads:[1,10,25]

$$\left[\begin{pmatrix} \mathbf{A}^{\mathrm{q}} & \mathbf{A}^{\mathrm{q}\mu} \\ \mathbf{T}^{\mu\mathrm{q}} & \mathbf{T}^{\mu\mu} \end{pmatrix} - \begin{pmatrix} z(\omega)\mathbf{I}_N & 0 \\ 0 & z'(\omega)\mathbf{I}_N \end{pmatrix}\right] \begin{pmatrix} \mathbf{q} \\ \boldsymbol{\mu} \end{pmatrix} = \begin{pmatrix} \mathbf{R} \\ -\mathbf{E}^{\mathrm{ext}} \end{pmatrix}. \tag{S1}$$

$$\begin{aligned} \mathbf{A}^{q}_{ij} &= \sum_{k=1}^{N} \overline{K}_{ik}(\mathbf{T}^{\mathrm{qq}}_{kj} - \mathbf{T}^{\mathrm{qq}}_{ij}) = (\overline{K} - P)\mathbf{T}^{\mathrm{qq}} \\ \mathbf{A}^{q\mu}_{ij} &= \sum_{k=1}^{N} \overline{K}_{ik}(\mathbf{T}^{\mathrm{q}\mu}_{kj} - \mathbf{T}^{\mathrm{q}\mu}_{ij}) = (\overline{K} - P)\mathbf{T}^{\mathrm{q}\mu} \\ \mathbf{R}_i &= \sum_{k=1}^{N} \overline{K}_{ik}(\mathbf{V}^{\mathrm{ext}}_{i} - \mathbf{V}^{\mathrm{ext}}_{k}) = -(\overline{K} - P)\mathbf{V}^{\mathrm{ext}} \end{aligned}$$

$$z(\omega) = -\mathrm{i}\frac{\omega}{w(\omega)}, \qquad z'(\omega) = -\frac{1}{\alpha^{\omega}}$$

where $\mathbf{T}^{\mathrm{qq}}$, $\mathbf{T}^{\mathrm{q}\mu}$, $\mathbf{T}^{\mu\mathrm{q}}$, and $\mathbf{T}^{\mu\mu}$ represent the charge–charge, charge–dipole, dipole–charge, and dipole–dipole kernels, respectively. The terms $z(\omega)$ and $z'(\omega)$ introduce the explicit frequency dependence for charges and dipoles, with α^{ω} the atomic polarizability at frequency ω. Finally, $\mathbf{V}^{\mathrm{ext}}$ and $\mathbf{E}^{\mathrm{ext}}$ are the external electrostatic potential and electric field evaluated at the atomic positions.[1,10,25]

S2.2 The ωFQFμ-BEM model

ωFQFμ-BEM extends the ωFQFμ model by describing part of the plasmonic substrate atomistically at the ωFQFμ level, while representing the remaining region as an implicit dielectric medium whose surface is discretized into small triangles (*tesserae*) and treated using the Boundary Element Method in the Integral Equation Formalism (BEM-IEF) for solving the polarizable continuum model equations.[2] In this framework, the ωFQFμ charges ($\mathbf{q}$) and dipoles ($\boldsymbol{\mu}$) mutually polarize with the BEM surface charges ($\boldsymbol{\sigma}$), modifying the response of the whole system.

For a system composed of N ωFQFμ atoms and T BEM *tesserae*, the resulting ωFQFμ-BEM equations read:[2]

$$\left[\begin{pmatrix} \mathbf{A}^{q} & \mathbf{A}^{q\mu} & \mathbf{A}^{q\sigma} \\ \mathbf{T}^{\mu q} & \mathbf{T}^{\mu\mu} & \mathbf{T}^{\mu\sigma} \\ \mathbf{B}^{\sigma q} & \mathbf{B}^{\sigma\mu} & \mathbf{B}^{\sigma} \end{pmatrix} - \begin{pmatrix} z(\omega)\mathbf{I}_N & 0 & 0 \\ 0 & z'(\omega)\mathbf{I}_N & 0 \\ 0 & 0 & \xi(\omega) \end{pmatrix}\right] \begin{pmatrix} \mathbf{q} \\ \mu \\ \sigma \end{pmatrix} = \begin{pmatrix} \mathbf{R} \\ -\mathbf{E}^{\text{ext}} \\ \mathbf{R_B} \end{pmatrix} \tag{S2}$$

where the different blocks are defined by the matrices:

$$\begin{aligned}
\mathbf{A}^{q}_{ij} &= \sum_{k=1}^{N} \overline{K}_{ik}(\mathbf{T}^{\text{qq}}_{kj} - \mathbf{T}^{\text{qq}}_{ij}) = (\overline{K} - P)\mathbf{T}^{\text{qq}} & \mathbf{B}^{\sigma q} &= \left(2\pi \mathbf{I}_T + \mathbf{F}\right)\mathbf{T}^{\sigma \text{q}} \\
\mathbf{A}^{q\mu}_{ij} &= \sum_{k=1}^{N} \overline{K}_{ik}(\mathbf{T}^{\text{q}\mu}_{kj} - \mathbf{T}^{\text{q}\mu}_{ij}) = (\overline{K} - P)\mathbf{T}^{\text{q}\mu} & \mathbf{B}^{\sigma\mu} &= \left(2\pi \mathbf{I}_T + \mathbf{F}\right)\mathbf{T}^{\sigma\mu} \\
\mathbf{A}^{q\sigma}_{ij} &= \sum_{k=1}^{N} \overline{K}_{ik}(\mathbf{T}^{\text{q}\sigma}_{kv} - \mathbf{T}^{\text{q}\sigma}_{iv}) = (\overline{K} - P)\mathbf{T}^{\text{q}\sigma} & \mathbf{B}^{\sigma} &= \mathbf{FSA}^{-1} \\
\mathbf{R}_{i} &= \sum_{k=1}^{N} \overline{K}_{ik}(\mathbf{V}^{\text{ext}}_{i} - \mathbf{V}^{\text{ext}}_{k}) = -(\overline{K} - P)\mathbf{V}^{\text{ext}} & \mathbf{R_B} &= -\left(2\pi \mathbf{I}_T + \mathbf{F}\right)\mathbf{V}^{\text{ext}}
\end{aligned}$$

$$z(\omega) = -\mathrm{i}\frac{\omega}{w(\omega)}, \qquad z'(\omega) = -\frac{1}{\alpha^{\omega}}, \qquad \xi(\omega) = -2\pi\frac{\varepsilon_2(\omega)+\varepsilon_1(\omega)}{\varepsilon_2(\omega)-\varepsilon_1(\omega)}\mathbf{I}_T\mathbf{SA}^{-1}$$

In Eq. (S2), $\mathbf{T}^{\text{qq}}$, $\mathbf{T}^{\text{q}\mu}$, and $\mathbf{T}^{\mu\mu}$ are the ωFQFμ charge-charge, charge-dipole, and dipole-dipole interaction kernels, respectively, whereas $\mathbf{T}^{\text{q}\sigma}$ and $\mathbf{T}^{\mu\sigma}$ describe the ωFQFμ-BEM charge-charge and dipole-charge couplings. The $\mathbf{S}$ and $\mathbf{F}$ matrices, in turn, depend on the geometry of the BEM *tesserae* and characterize the BEM response.[2,28,29]

S2.3 Multiscale TDDFT/ωFQFμ (BEM)

To describe fluorescence modifications induced by a nearby silver tip, we employed a multiscale Time-Dependent Density Functional Theory (TDDFT)/ωFQFμ framework recently developed by some of the present authors. Within this scheme, the standard Casida equations are extended to include the polarization response of the metallic environment as:[4,30]

$$\left[\begin{pmatrix} \mathbf{A}(\omega) & \mathbf{B}(\omega) \\ \mathbf{B}^*(-\omega^*) & \mathbf{A}^*(-\omega^*) \end{pmatrix} - \omega \begin{pmatrix} \mathbf{I} & 0 \\ 0 & -\mathbf{I} \end{pmatrix}\right] \begin{pmatrix} \mathbf{X}_\omega \\ \mathbf{Y}_\omega \end{pmatrix} = \mathbf{0} \tag{S3}$$

where ω is the excitation frequency and $\mathbf{X}_\omega/\mathbf{Y}_\omega$ are the corresponding excitation/de-excitation transition densities, respectively. The characteristic Casida matrix blocks read:[4,31]

$$A_{ai,bj}(\omega) = (\varepsilon_a - \varepsilon_i)\delta_{ab}\delta_{ij} + K^0_{ai,bj} + K^{\text{pol}}_{ai,bj}(\omega) \tag{S4a}$$

$$B_{ai,bj}(\omega) = K^0_{ai,jb} + K^{\text{pol}}_{ai,jb}(\omega) \tag{S4b}$$

where ε_i and ε_a are the occupied and virtual orbital energies, respectively, and $K^0_{ai,bj}$ is the exchange–correlation kernel. $\mathbf{K}^{\text{pol}}(\omega)$ accounts for the environment polarization arising from the ωFQFμ metallic tip, through its induced charges $\text{q}(\omega)$ and dipoles $\mu(\omega)$, as:[2,4,32]

$$\begin{aligned} \text{K}^{\text{pol}}_{ai,bj}(\omega) = &\sum_{\nu} \text{q}(\omega)(r_\nu, [\Psi^*_a\Psi_i])\mathbf{V}(r_\nu, [\Psi^*_b\Psi_j]) \\ &- \sum_{\nu} \mu(\omega)(r_\nu, [\Psi^*_a\Psi_i])\mathbf{E}(r_\nu, [\Psi^*_b\Psi_j]) \end{aligned} \tag{S5}$$

where $\Psi^*_a\Psi_i$ represents the distribution associated with the $(i \rightarrow a)$ excitation inducing the classical ωFQFμ charges and dipoles. These, in turn, polarize the quantum subsystem through their interaction with the electrostatic potential $\mathbf{V}(r_\nu, [\Psi^*_b\Psi_j])$ and electric field $\mathbf{E}(r_\nu, [\Psi^*_b\Psi_j])$, both evaluated at the atomic position r_ν.[4,31]

After solving Eq. (S3), the rates of excitation, radiative decay, and non-radiative decay are computed

as:[4,31,33,34]

$$\Gamma_{\text{ex}} = \frac{2\pi}{3c}|\mathbf{d}_{\text{tot}}^{0\text{K}}|^2 \tag{S6}$$

$$\Gamma_{\text{rad}} = \frac{4\omega'^3}{3c^3}|\mathbf{d}_{\text{tot}}^{\text{K}0}|^2 \tag{S7}$$

$$\Gamma_{\text{nr}} = -2\omega'' \tag{S8}$$

where c represents the speed of light, ω'/ω'' are the computed real/imaginary frequencies, and $\mathbf{d}_{\text{tot}}^{0\text{K}}$ is the total transition dipole moment for the $0 \rightarrow \text{K}$ transition.

As a final remark, for a pure BEM or mixed ωFQFμ-BEM description of the plasmonic substrate, the same formalism applies, except that $\mathbf{K}^{\text{pol}}(\omega)$ also includes the contribution from the induced BEM surface charges $\boldsymbol{\sigma}(\omega)$ interacting with the electrostatic potential ($\mathbf{V}(r_V, [\Psi_b^*\Psi_j])$, describing the polarization of the continuum part of the metallic environment.

S2.4 Spectral Overlap

The spectral overlap between Platinum-Phthalocyanine (PtPc) and Zinc-Phthalocyanine (ZnPc) molecules is calculated following the approach reported in Cao *et al.*, *Nat. Chem.* **13**, 766–770 (2021).[35] Specifically, the fluorescence spectra of PtPc and ZnPc are digitized from Fig. 1c of Ref. 9. Then, the absorption spectrum of ZnPc was generated by inverting the emission spectrum around its intensity maximum. The spectral overlap integral (J) is then calculated as:[35]

$$J = \int_{E_1}^{E_2} F_{\text{D}}(E)\,\varepsilon_{\text{A}}(E)\,\text{d}E,$$

where F_{D} and ε_{A} are the donor fluorescence and acceptor absorption spectra, respectively, both normalized as:

$$\int_{E_1}^{E_2} F_{\text{D}}(E)\,\text{d}E = 1 \qquad \int_{E_1}^{E_2} \varepsilon_{\text{A}}(E)\,\text{d}E = 1$$

Fig. S1 shows the normalized emission and absorption spectra used for the spectral overlap calculation. The resulting spectral overlap integral is $J = 2.968 \times 10^{-2}$ eV^{-1}.

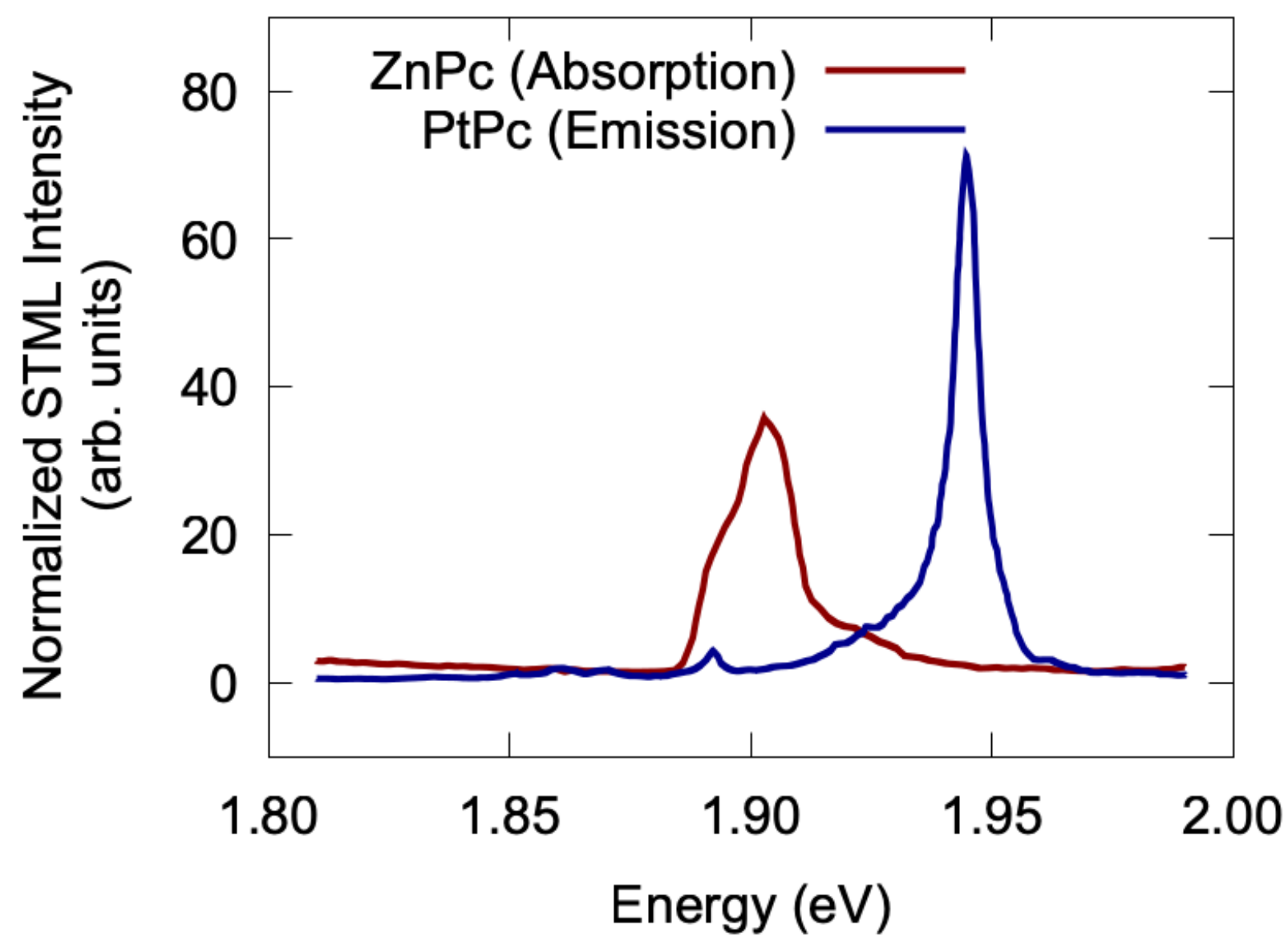

Figure S1: Normalized STML emission spectrum of PtPc (donor) and the reconstructed absorption spectrum of ZnPc (acceptor). Data adapted with permission from Ref. 9. Copyright 2022 Springer Nature.

S2.5 EET-mediated TDDFT/ωFQFμ Fluorescence Emission

We calculate the electronic energy transfer (EET) rate from the i-th donor excited state to the j-th acceptor excited state, $\Gamma_{\mathrm{EET}}^{i\to j}$, using Fermi's golden rule as:[6,36]

$$\Gamma_{\mathrm{EET}}^{i\to j} = \frac{2\pi}{\hbar} |V_{ij}|^2 J, \tag{S9}$$

where V_{ij} is the electronic coupling between states i and j, $\hbar$ is the reduced Planck constant, and J is the donor–acceptor spectral overlap computed from experimental spectra.[9,35]

To compute V_{ij}, we neglect exchange–correlation and density-overlap contributions associated with Dexter-type mechanisms, which are expected to be negligible at the donor–acceptor separations considered in the present study.[6,9,36] Under this assumption, energy transfer is treated as a purely Förster Resonance

Energy Transfer (FRET)-like process, and V_{ij} is evaluated as:[6,36]

$$
\begin{aligned}
V_{ij} &= V_{ij}^{\text{Coulomb}} + V_{ij}^{\text{Environment}} \\
&= \int d\mathbf{r}\, d\mathbf{r}' \, \frac{\tilde{\rho}^*_{A,j}(\mathbf{r})\, \tilde{\rho}_{D,i}(\mathbf{r}')}{|\mathbf{r}-\mathbf{r}'|} \\
&\quad + \sum_{v} \left(\int d\mathbf{r} \, \frac{\tilde{\rho}^*_{A,j}(\mathbf{r})}{|\mathbf{r}-\mathbf{r}_v|} \right) \mathrm{q}(\omega)(\mathbf{r}_v, \tilde{\rho}_{D,i}) \\
&\quad - \sum_{v} \left(\int d\mathbf{r} \, \frac{\mathbf{r}-\mathbf{r}_v}{|\mathbf{r}-\mathbf{r}_v|^3} \right) \tilde{\rho}^*_{A,j}(\mathbf{r}) \vec{\mu}(\omega)(\mathbf{r}_v, \tilde{\rho}_{D,i}).
\end{aligned} \tag{S10}
$$

Here, $\tilde{\rho}_{D,i}(\mathbf{r}')$ and $\tilde{\rho}^*_{A,j}(\mathbf{r})$ are the tip-perturbed transition densities of the donor and acceptor for the i-th and j-th excited states, respectively. The quantities $\mathrm{q}(\omega)(\mathbf{r}_v, \tilde{\rho}_{D,i})$ and $\vec{\mu}(\omega)(\mathbf{r}_v, \tilde{\rho}_{D,i})$ denote the ωFQFμ fluctuating charges and dipoles at atomic site $\mathbf{r}_v$ resonant with frequency ω,[1,10] and induced by the donor transition density.[4] For ωFQFμ-BEM calculations,[2] a term accounting for the polarization of the BEM $\sigma(\omega)$ charges must be added in Eq. (S10).

The fluorescence emission intensities of the donor and acceptor are then evaluated as follows:[6]

$$
I^i_{\mathrm{D}} = \Gamma^i_{\mathrm{ex,D}} \cdot \Phi^i_{\mathrm{D}} = \Gamma^i_{\mathrm{ex,D}} \cdot \frac{\Gamma^i_{\mathrm{rad,D}}}{\sum_j \Gamma^{i\to j}_{\mathrm{EET}} + \Gamma^i_{\mathrm{rad,D}} + \Gamma^i_{\mathrm{nr,met,D}} + \Gamma_{\mathrm{nr,0,D}}}, \tag{S11a}
$$

$$
\begin{aligned}
I^i_{\mathrm{A}} &= \Gamma^i_{\mathrm{ex,D}} \sum_j \eta^{i\to j}_{\mathrm{EET}} \cdot \Phi^j_{\mathrm{A}} \\
&= \Gamma^i_{\mathrm{ex,D}} \sum_j \frac{\Gamma^{i\to j}_{\mathrm{EET}}}{\Gamma^{i\to j}_{\mathrm{EET}} + \Gamma^i_{\mathrm{rad,D}} + \Gamma^i_{\mathrm{nr,met,D}} + \Gamma^i_{\mathrm{nr,0,D}}} \cdot \frac{\Gamma^j_{\mathrm{rad,A}}}{\Gamma^j_{\mathrm{rad,A}} + \Gamma^j_{\mathrm{nr,met,A}} + \Gamma^j_{\mathrm{nr,0,A}}},
\end{aligned} \tag{S11b}
$$

where $\Gamma^i_{\mathrm{ex},X}$ is the excitation rate into state i, $\Gamma^i_{\mathrm{rad},X}$ and $\Gamma^i_{\mathrm{nr,met},X}$ are the radiative and non-radiative decay rates, respectively, and $\Gamma_{\mathrm{nr},0,X}$ is the intrinsic non-radiative decay rate of the molecule, with $X \in \{\mathrm{D}, \mathrm{A}\}$ (donor, acceptor). Φ^i_X denotes the fluorescence quantum yield, and $\eta^{i\to j}_{\mathrm{EET}}$ is the EET efficiency from the donor state i to the acceptor state j, defined as the fraction of the donor's total decay rate that proceeds via EET: $\eta^{i\to j}_{\mathrm{EET}} \to 1$ when the EET rate $\Gamma^{i\to j}_{\mathrm{EET}}$ dominates over the donor's other decay channels, and $\eta^{i\to j}_{\mathrm{EET}} \to 0$ when the probability of energy transfer is negligible compared to the donor's other decay channels.

The STM tunneling excitation process is modeled consistently with the negative bias voltage applied in the experiment.[9] Under these conditions, electrons are withdrawn from the HOMO of each chromophore

by the tip and subsequently injected from the substrate into either the LUMO or LUMO+1 with equal probability.[6,37] This assumption is justified by the degeneracy of the LUMO and LUMO+1 in both PtPc and ZnPc, as shown in Fig. S2a,b. Tunneling excitation is applied only to the donor, given the close tip–donor proximity in our simulations (5 Å), within the sub-nanometer range over which STM tunneling current is appreciable. Following on this basis, the acceptor's own excitation rate is not included, and it is excited exclusively due to the EET process. The acceptor's radiative and non-radiative decay rates, however, are still evaluated in the presence of the tip and enter its fluorescence quantum yield. Within this framework, the total fluorescence emission comprises two contributions: (i) direct tip-perturbed PtPc emission from its S_1 (HOMO$\rightarrow$LUMO) and S_2 (HOMO$\rightarrow$LUMO+1) excited states, and (ii) EET-mediated ZnPc emission, which reflects both the coupling between each PtPc excited state i and each ZnPc excited state j and the tip-perturbed decay properties of ZnPc itself. The transition dipoles associated with these transitions and their relative orientations in the simulated geometry are depicted in Fig. S2c.

S2.6 FRET Fit from Experimental Data

To compare the computed EET-mediated ZnPc fluorescence intensity decay with the experimental reference, we digitized the ZnPc intensity data from Fig. 2b of Ref. 9 in the FRET regime ($d \geq 1.72$ nm). The data were fitted using the functional form reported in the same experimental work:

$$I_{\mathrm{ZnPc}}(d) = \frac{1}{A + Bd^6}, \tag{S12}$$

with $A = -1.47 \times 10^{-2}\, I^{-1}$ and $B = 1.70 \times 10^{-2}\, I^{-1}\,\mathrm{nm}^{-6}$. The resulting FRET fitting curve is included in Fig. 4 of the main text.

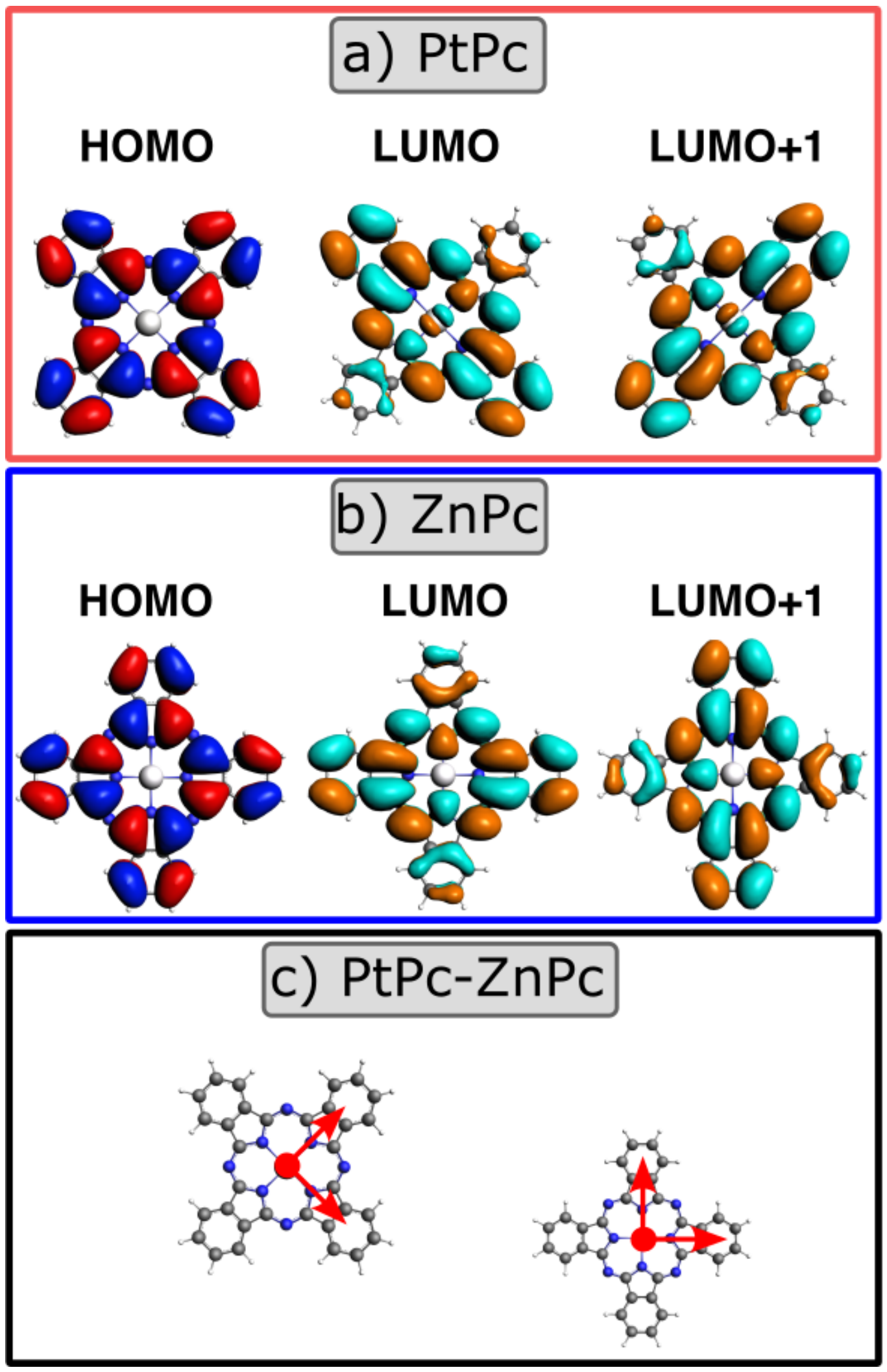

Figure S2: Frontier molecular orbitals of PtPc (a) and ZnPc (b): HOMO (left), LUMO (center), and LUMO+1 (right), computed in vacuum at the CAM-B3LYP/DZP level of theory. (c) Schematic representation of the PtPc–ZnPc geometry used in the simulations, with red arrows indicating the transition dipoles associated with the S_1 and S_2 excited states of PtPc and ZnPc.

S3 Results

S3.1 Molecular structures and tips optical response

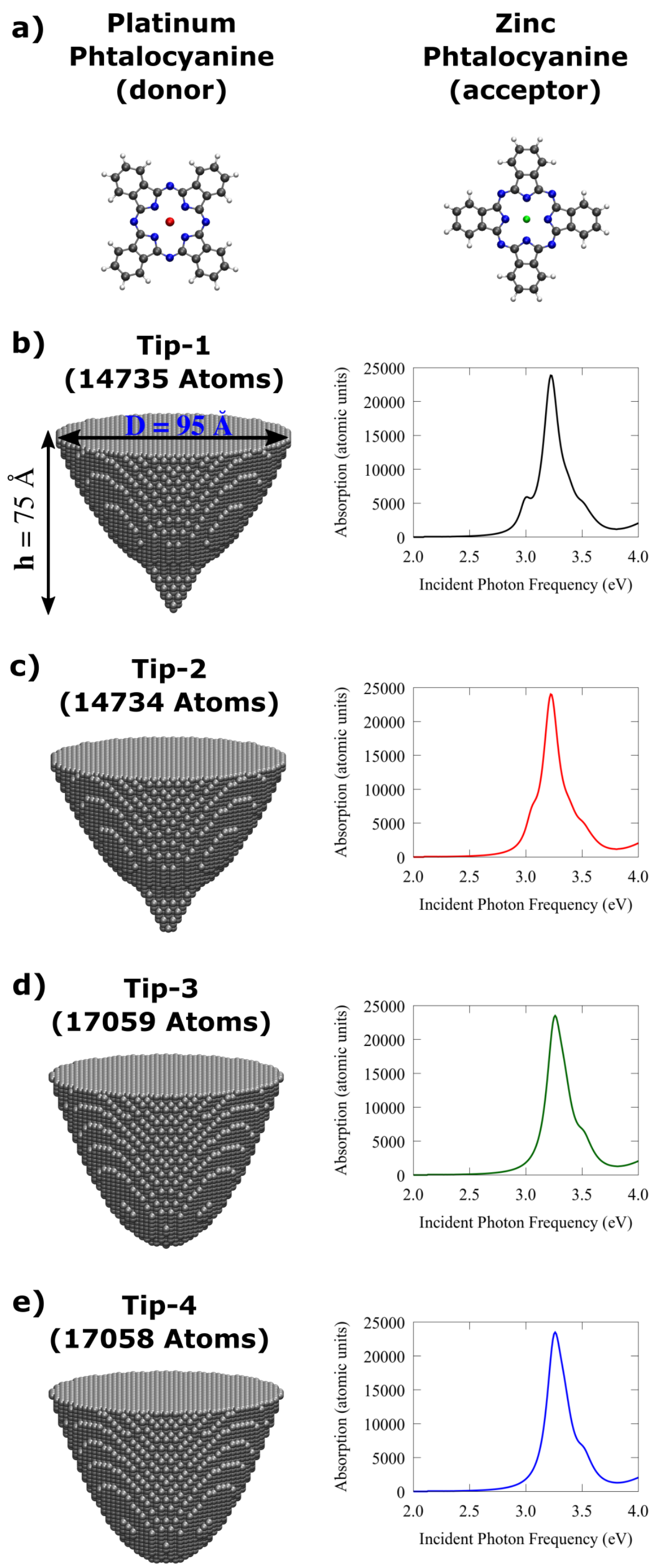

Figure S3: Molecular structures of PtPc and ZnPc (a). Tip-1 (b), Tip-2 (c), Tip-3 (d), and Tip-4 (e) geometries (left) and their computed ωFQFμ absorption spectra (right).

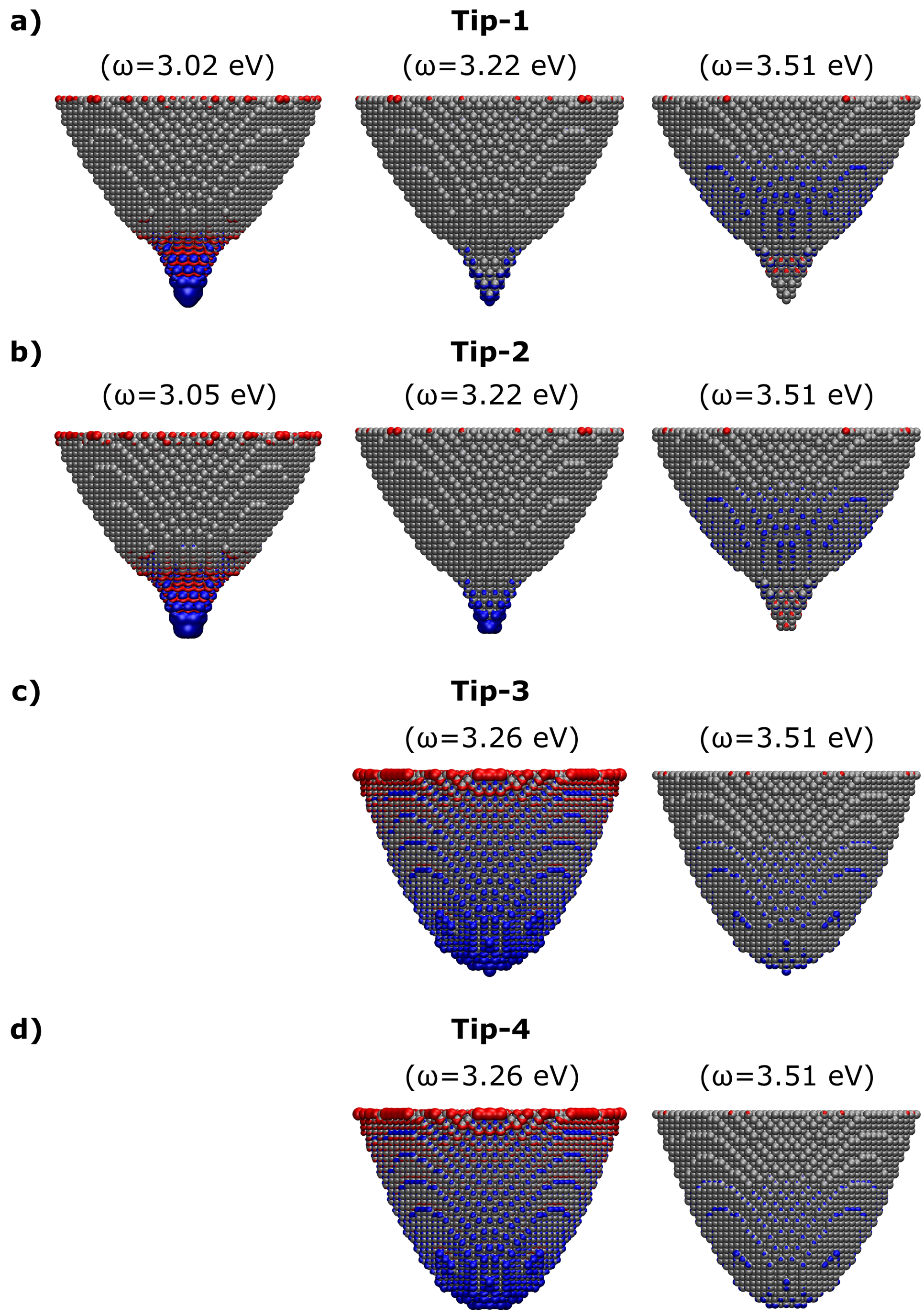

Figure S4: Induced ωFQFμ density under a z-polarized electric field for (a) Tip-1 and (b) Tip-2, evaluated at the resonant frequencies of the apex–edge plasmon modes. Isovalues are set to 10^{-5}.

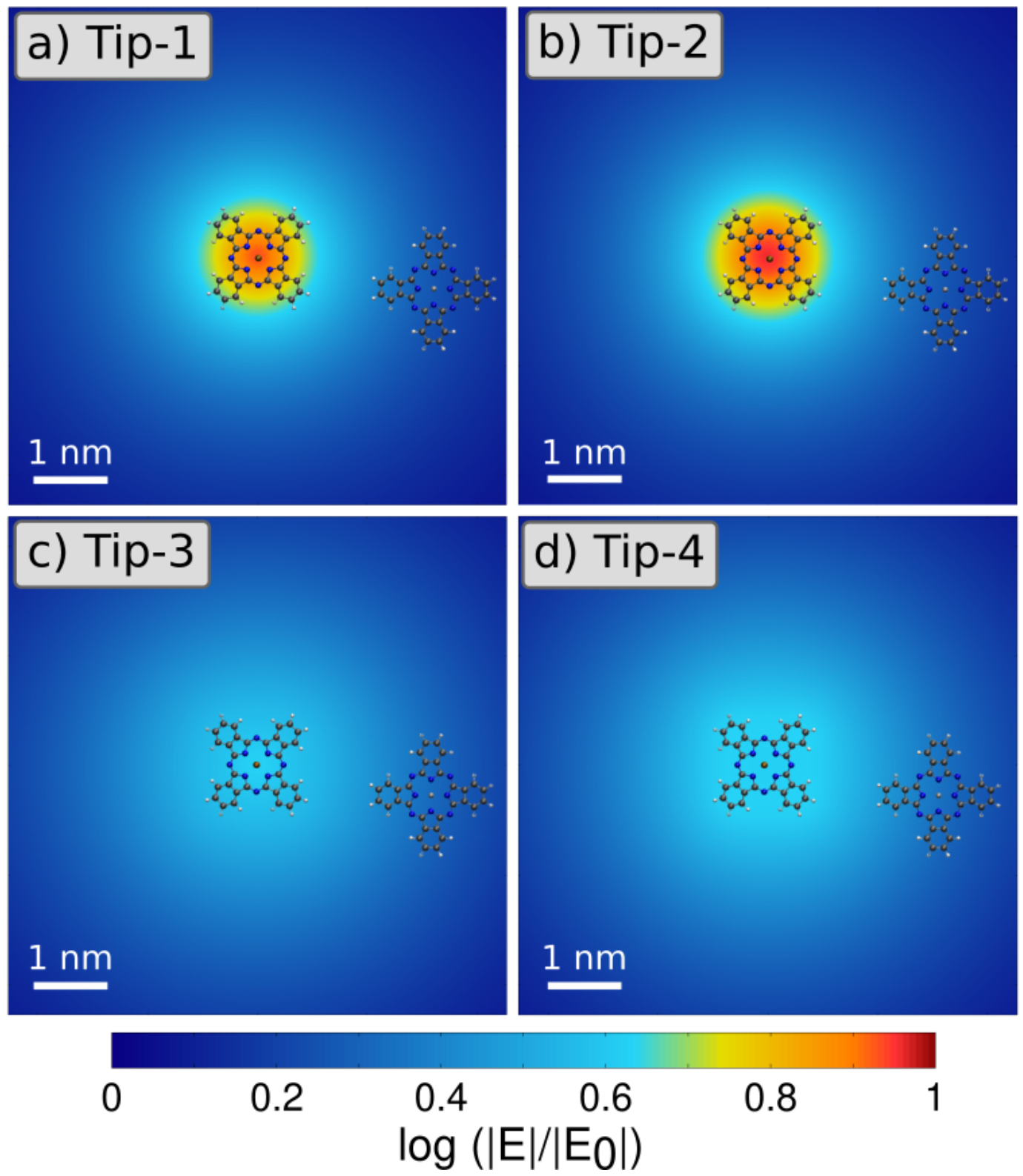

Figure S5: ωFQFμ electric-field enhancement maps (simulation frequency $\omega = 2.5$ eV) for (a) Tip-1, (b) Tip-2, (c) Tip-3, and (d) Tip-4 positioned at 5 Å above PtPc (site C), calculated on the molecular plane (PtPc and ZnPc geometries are also displayed).

In Fig. S5, we depict the electric-field enhancement maps generated by the four simulated tips placed 5 Å above PtPc (site C) on the XY plane containing the molecules, under a z-polarized monochromatic external field at ω = 2.5 eV, matching the frequency used in the TDDFT/ωFQFμ simulations. Tip-1 produces an enhanced field localized mainly around the Pt center (Fig. S5a), while Tip-2 exhibits a more spatially extended high-field region on the molecular plane (Fig. S5b). Tip-3 yields the weakest field enhancement overall (Fig. S5c), whereas Tip-4 displays a slightly stronger enhancement than Tip-3 (Fig. S5d). Specifically, removing the apex atom from Tip-1 to Tip-2 and from Tip-3 to Tip-4 broadens the field distribution and increases its intensity at the tip–molecule separation considered in this work (5 Å), consistent with the broadening reported for similar apex modifications,[38,39] whereas the larger morphological change from Tip-1/Tip-2 to Tip-3/Tip-4 reduces the field enhancement by more than 50%. We note that the effect of apex-atom removal on the field intensity is not universal and depends on the tip–molecule distance considered.[39] Despite these opposite effects on the local field enhancement distribution and magnitude, both single-atom and full morphological modifications comparably suppress the EET-driven fluorescence at site C, as compared with

Tip-1. These results cannot be rationalized in terms of simplified models based on the simulation of the electric field enhancement. Indeed, our findings underscore the need for a fully atomistic description of the plasmonic tip to capture how subtle apex rearrangements affect intermolecular EET, fully integrated with a quantum mechanical description of the molecular system.[4,32]

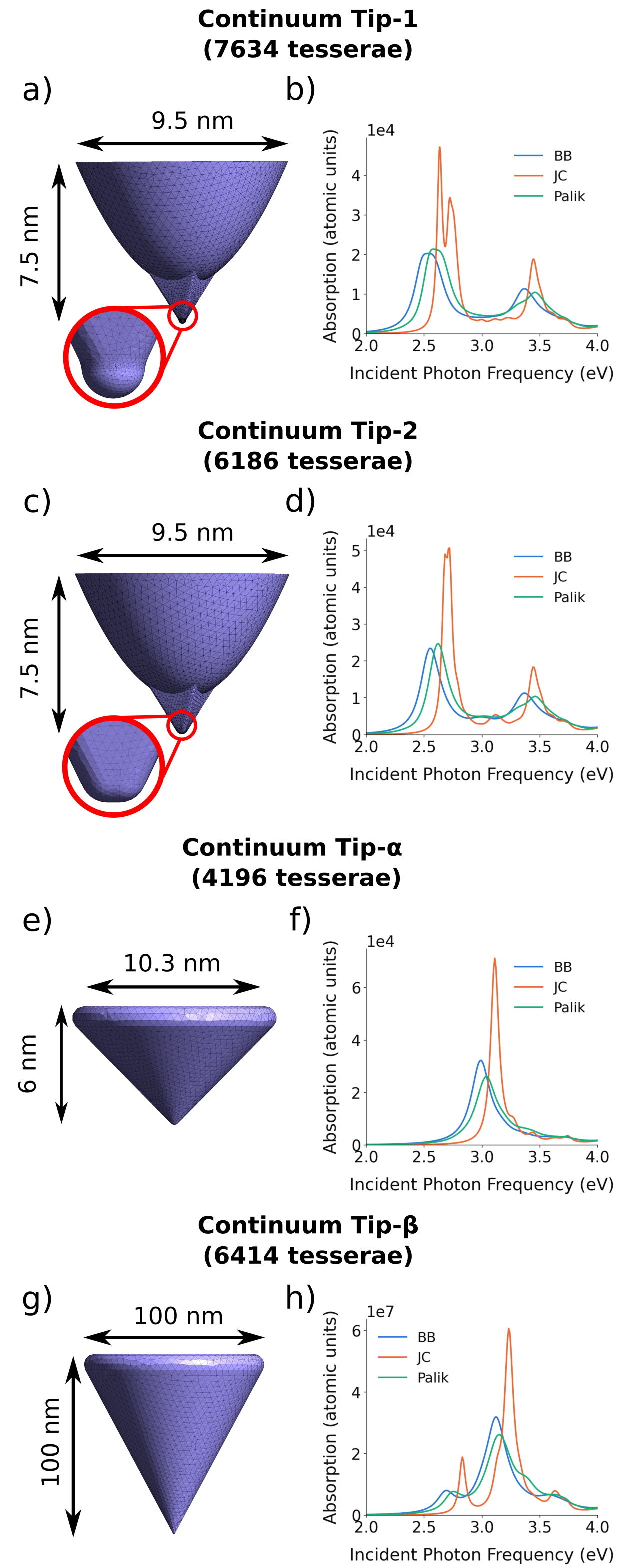

Figure S6: Continuum Tip-1 (a), Continuum Tip-2 (c), Continuum Tip-α (e) and Continuum Tip-β (g) geometries, along with their BEM-computed absorption spectra using the Dipolar Polarizable Continuum Model (b-d).The absorption spectrum is reported for each structure using the Brendel-Bormann (BB),[14] Johnson and Christy (JC),[15] and Palik[16] permittivity functions for silver.

S3.2 TDDFT/ωFQFμ (BEM) simulations

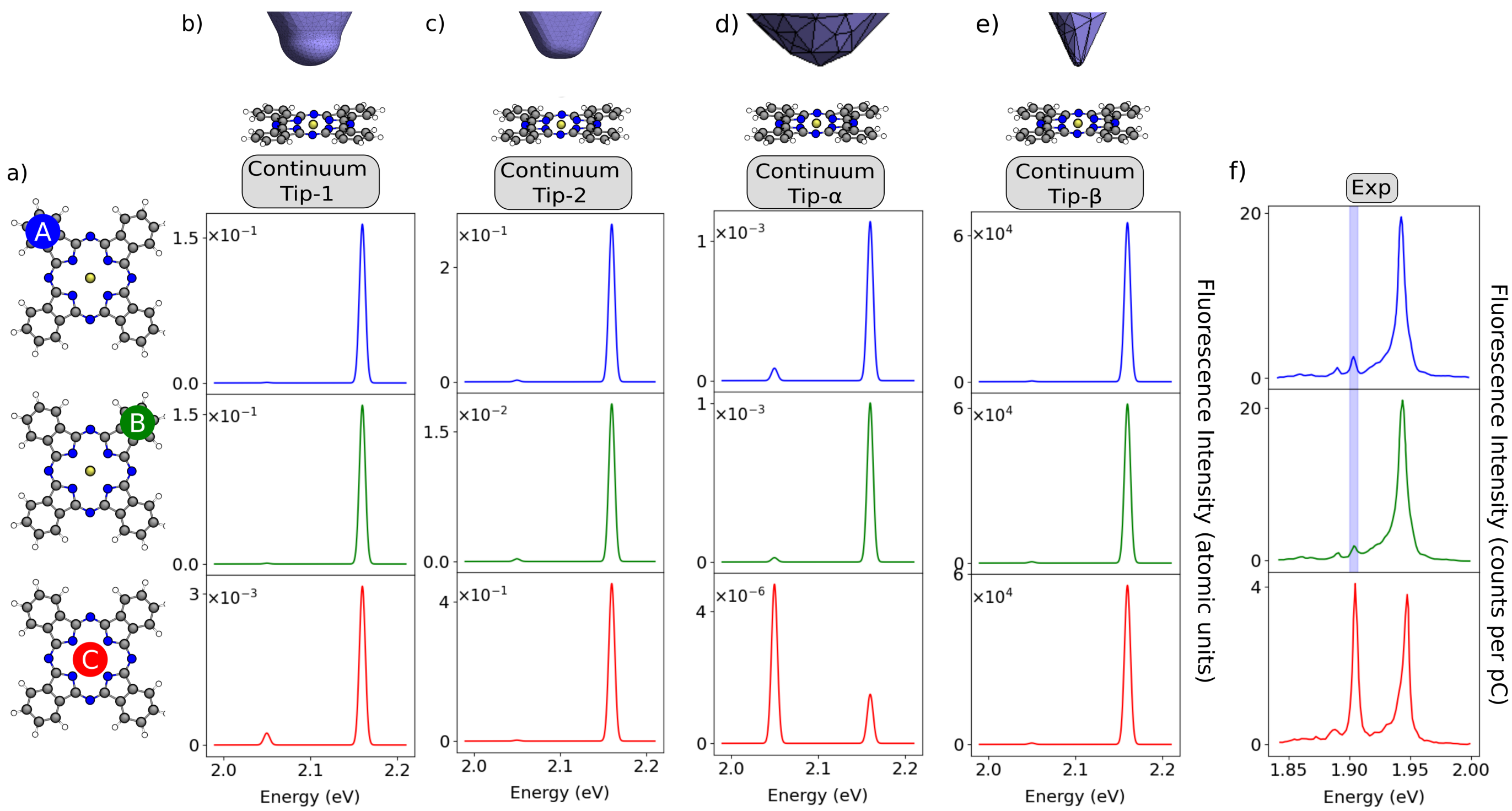

Figure S7: (a) Molecular geometry of PtPc, with the three tip positions A, B, and C highlighted. (b–e) Schematic depictions of Continuum Tip-1, Continuum Tip-2, Continuum Tip-α, and Continuum Tip-β, and the corresponding absolute TDDFT/BEM fluorescence spectra at positions A (blue), B (green), and C (red) (bottom). (f) Experimental fluorescence spectra reproduced with permission from from Ref. 9. Copyright 2022 Springer Nature; the shaded region highlights the PtPc emission band.

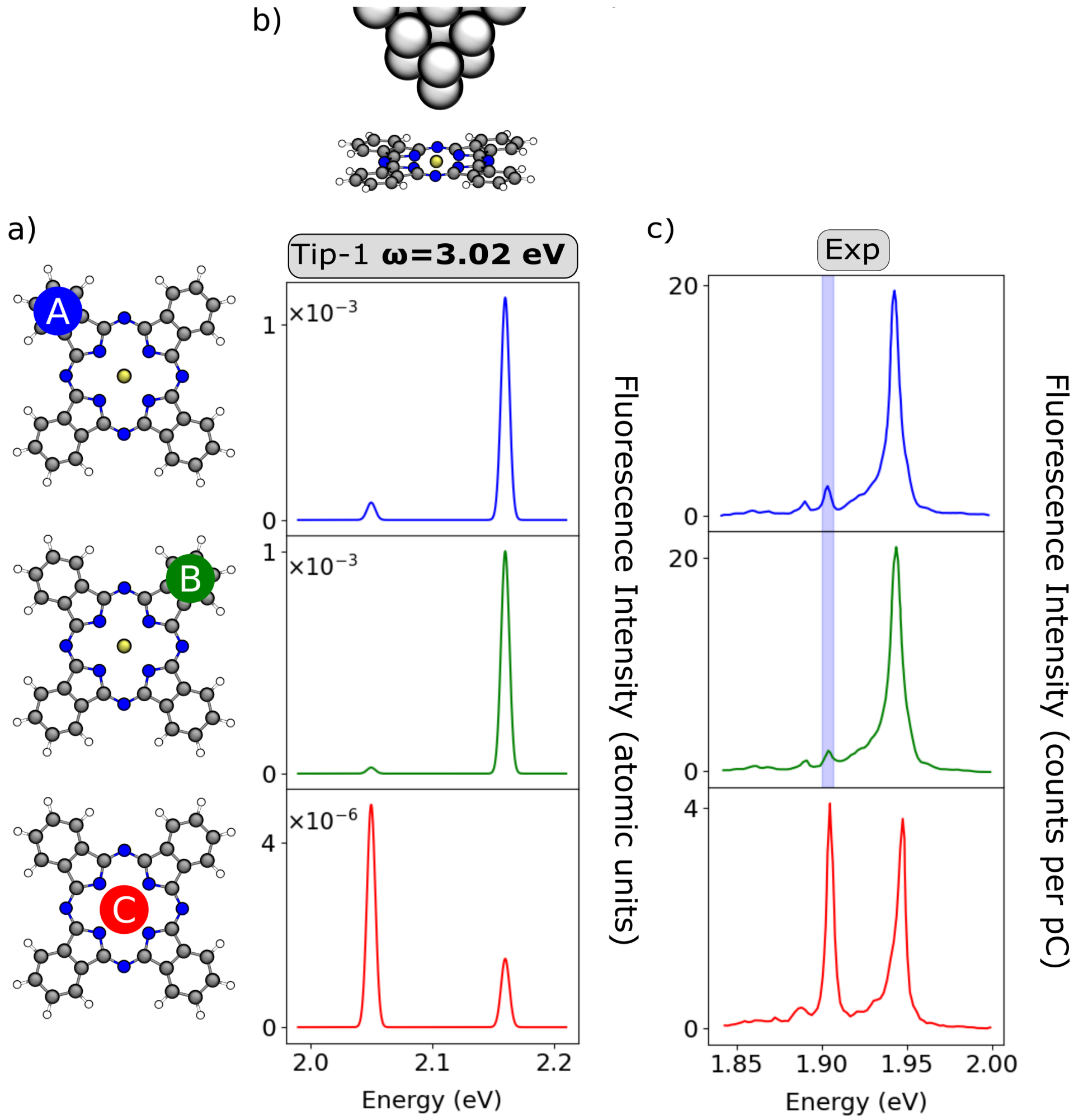

Figure S8: (a) Molecular geometry of PtPc, with the three tip positions A, B, and C highlighted. (b) Schematic depictions of Tip-1 (top), and the corresponding absolute TDDFT/ωFQFμ fluorescence spectra at positions A (blue), B (green), and C (red) (bottom), with the PRF (ω = 3.02 eV) as the simulation frequency. (c) Experimental fluorescence spectra reproduced with permission from Ref. 9. Copyright 2022 Springer Nature; the shaded region highlights the PtPc emission band.

Table S1: Absolute fluorescence intensity of the ZnPc (I_{ZnPc}) and PtPc (I_{PtPc}) emission peaks at tip positions A, B, and C, for the four simulated atomistic tip geometries and the four simulated continuum tip geometries, compared with the experimental values taken from Ref. 9

Dataset	Position	I_{ZnPc}	I_{PtPc}
Experiment (counts per pC)	A	2.59	19.6
	B	1.87	21.0
	C	4.08	3.80
Tip-1 (a.u.)	A	8.86×10^{-3}	1.28×10^{-1}
	B	6.78×10^{-3}	1.27×10^{-1}
	C	5.32×10^{-4}	4.99×10^{-4}
Tip-2 (a.u.)	A	1.03×10^{-2}	1.66×10^{-1}
	B	8.64×10^{-3}	1.66×10^{-1}
	C	1.26×10^{-4}	1.04×10^{-4}
Tip-3 (a.u.)	A	3.65×10^{-5}	7.84×10^{-4}
	B	1.85×10^{-5}	7.84×10^{-4}
	C	3.61×10^{-7}	5.25×10^{-7}
Tip-4 (a.u.)	A	1.19×10^{-5}	5.11×10^{-4}
	B	3.26×10^{-6}	5.12×10^{-4}
	C	1.20×10^{-8}	2.03×10^{-8}
Continuum Tip-1 (a.u.)	A	6.18×10^{-4}	1.64×10^{-1}
	B	7.93×10^{-4}	1.59×10^{-1}
	C	2.35×10^{-4}	3.14×10^{-3}
Continuum Tip-2 (a.u.)	A	2.93×10^{-3}	2.74×10^{-1}
	B	3.06×10^{-4}	1.82×10^{-2}
	C	2.69×10^{-3}	4.52×10^{-1}
Continuum Tip-α (a.u.)	A	8.91×10^{-5}	1.14×10^{-3}
	B	2.73×10^{-5}	1.00×10^{-3}
	C	4.82×10^{-6}	1.49×10^{-6}
Continuum Tip-β (a.u.)	A	3.28×10^{2}	6.52×10^{4}
	B	5.11×10^{2}	6.17×10^{4}
	C	4.71×10^{2}	5.61×10^{4}

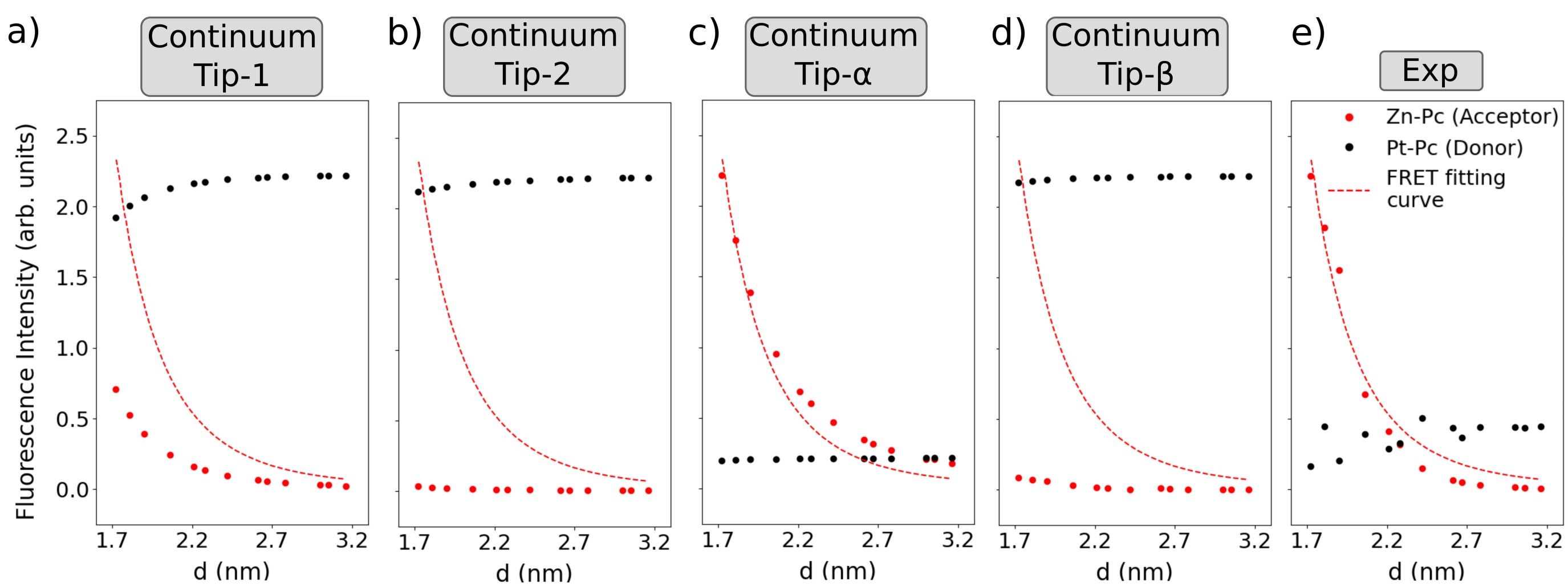

Figure S9: TDDFT/BEM PtPc and ZnPc fluorescence intensities as a function of the donor-acceptor intermetal distance for Continuum Tip-1, Continuum Tip-2, Continuum Tip-α and Continuum Tip-β, with the tip positioned at site C (right), compared with the experimental data reproduced with permission from Ref. 9. Copyright 2022 Springer Nature. (left). The simulated curve is normalized to the experimental ZnPc intensity at ($d = 1.72$) nm. Dashed lines represent the FRET fitting curve fit to the experimental data.

To evaluate the effect of the simulation frequency on the TDDFT/BEM calculations, Fig. S9 reports the RET_{eff} values of the four continuum tips as a function of distance, computed at $\omega = 2.0$ eV. Interestingly, this more off-resonant (red-shifted) frequency improves the agreement of Continuum Tip-1 with experiment, whereas Continuum Tip-2 still largely underestimates RET_{eff} at all distances. Continuum Tip-α and Continuum Tip-β, instead, respectively overestimate and underestimate RET_{eff}, with profiles qualitatively similar to those obtained at $\omega = 2.5$ eV. Therefore, tuning the simulation frequency can improve the agreement for a specific continuum geometry, but does not yield a consistent description across tip models, further supporting the need for an atomistic description of the tip, which becomes particularly critical when modelling single-atom modifications altering the EET response.

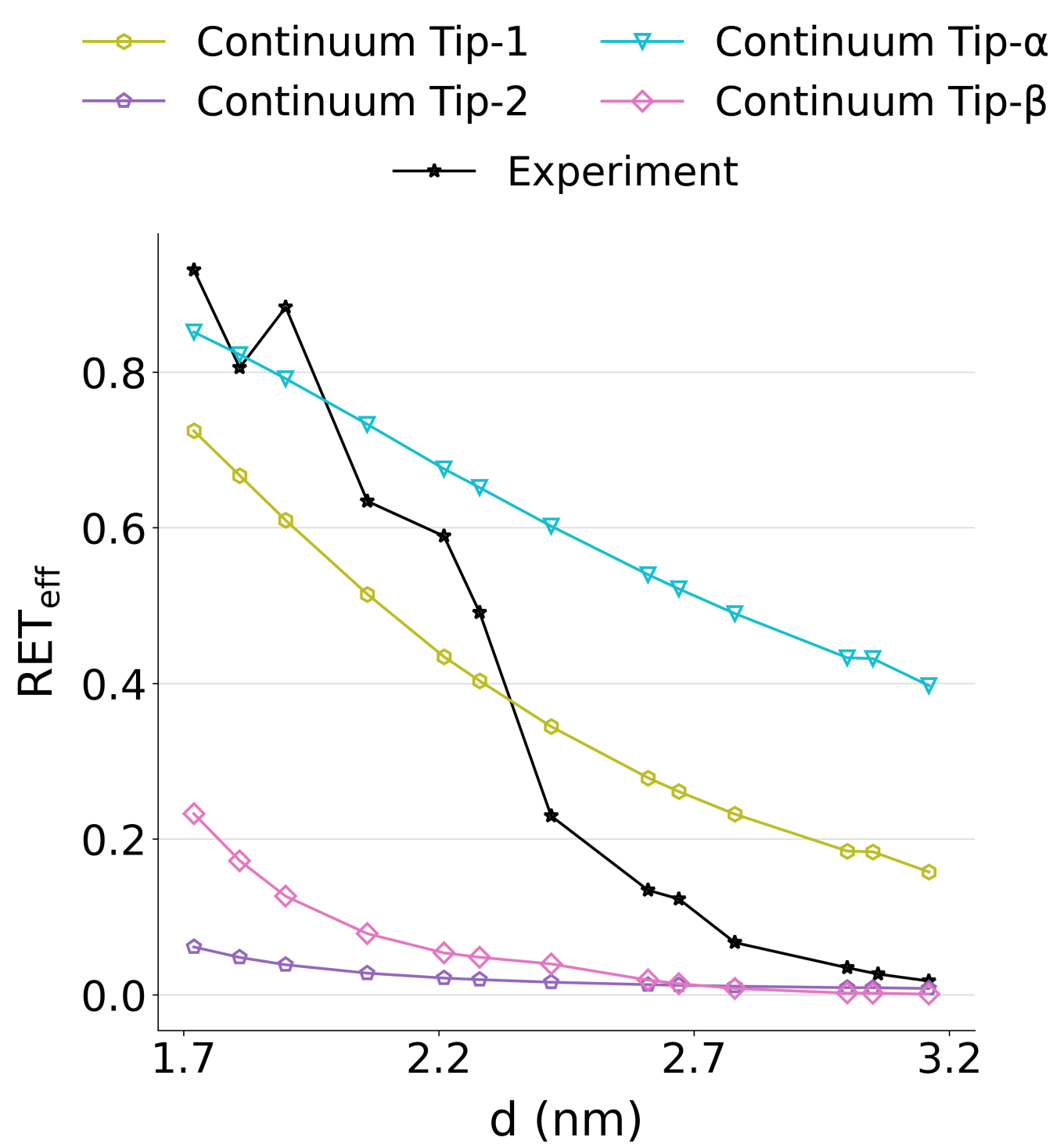

Figure S10: RET efficiency (simulation frequency: $\omega = 2.0$ eV) as a function of the donor-acceptor intermetal distance, with the tip positioned at site C, for the the four continuum tip models and the experimental reference (adapted with permission from Ref. 9. Copyright 2022 Springer Nature.).

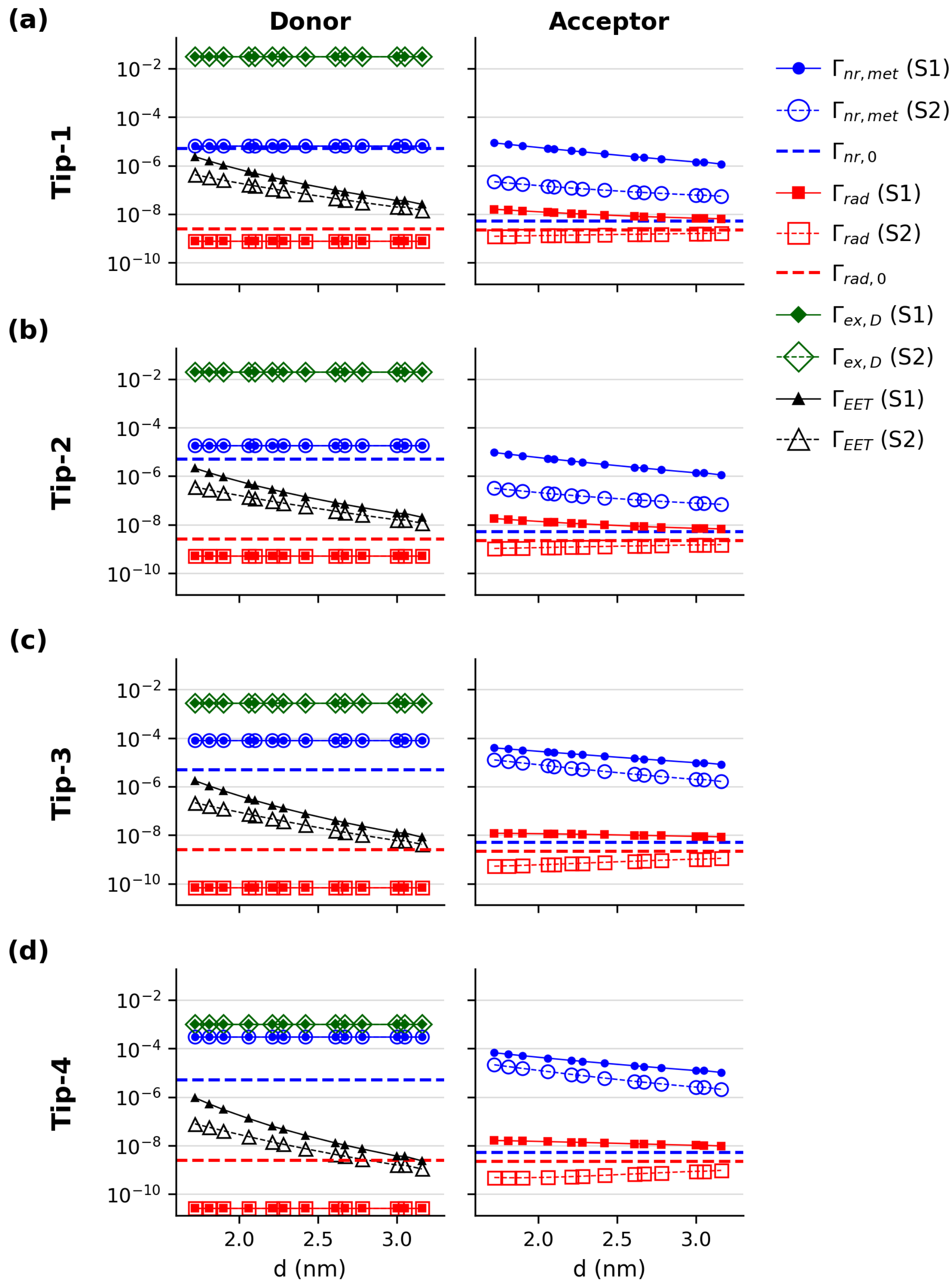

Figure S11: Non-radiative decay (blue), radiative decay (red), excitation (green), and EET (black) rates for the donor and acceptor molecules as a function of the donor–acceptor distance, for Tip-1 (a), Tip-2 (b), Tip-3 (c), and Tip-4 (d).

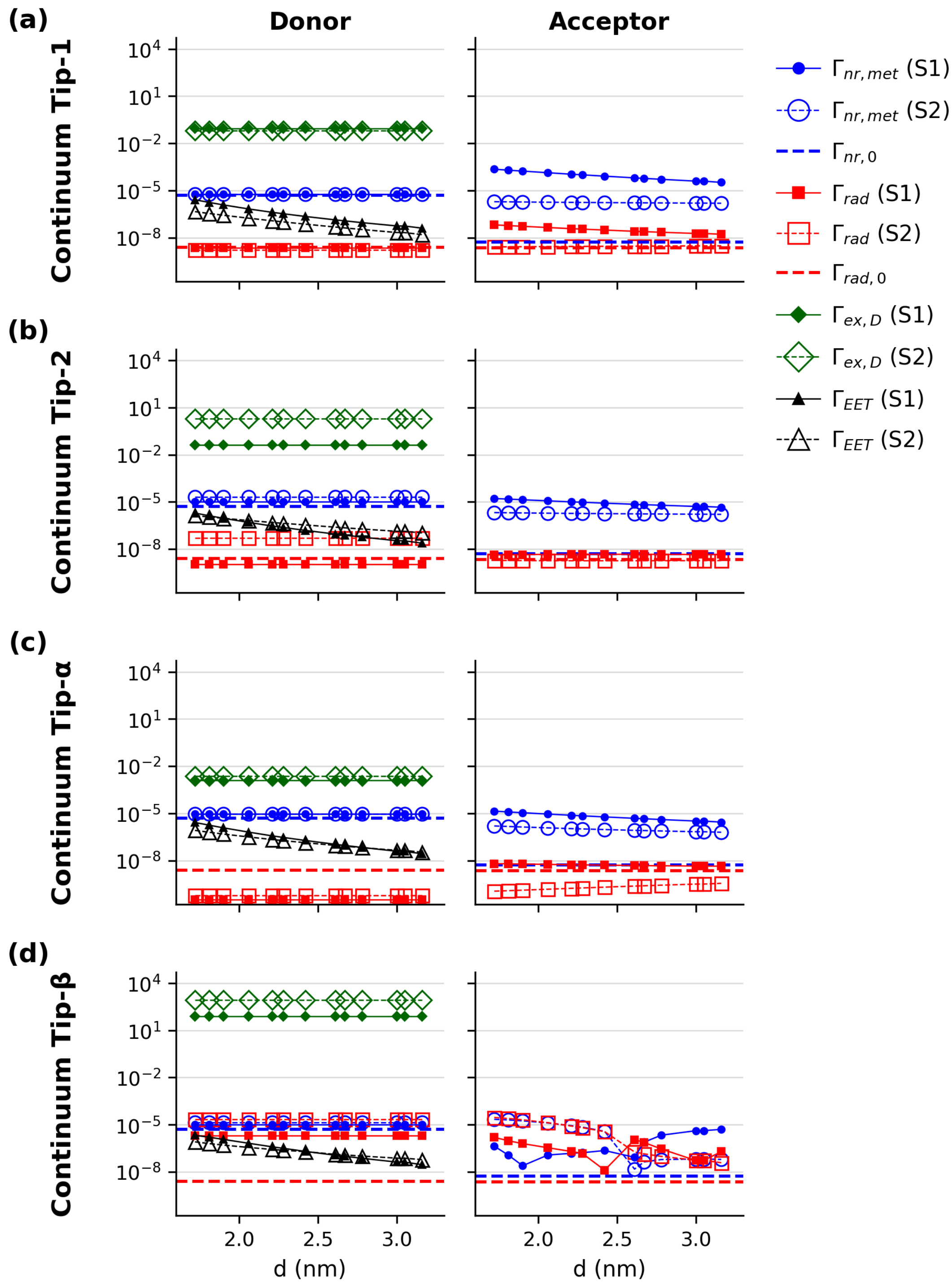

Figure S12: Non-radiative decay (blue), radiative decay (red), excitation (green), and EET (black) rates for the donor and acceptor molecules as a function of the donor–acceptor distance, for Continuum Tip-1 (a), Continuum Tip-2 (b), Continuum Tip-α (c), and Continuum Tip-β (d).

S3.3 Tip1-base simulations

To evaluate the effect of the silver slab acting as the experimental base, we first evaluated the tip-base response using the ωFQFμ-BEM formalism developed by some of the present authors[2] (see Section S2.2 for an explanation of the ωFQFμ-BEM formalism). Here, Tip-1 is treated in a full atomistic ωFQFμ manner and the experimental silver slab is modeled as a continuum BEM cube with rounded, smooth edges, reducing the computational cost while maintaining accuracy. In Fig. S13 we present a depiction of the Tip1-base ωFQFμ-BEM geometry setup studied initially, and the computed absorption spectrum for the ωFQFμ Tip-1 alone, the BEM base alone, and the combined Tip1-base ωFQFμ-BEM system.

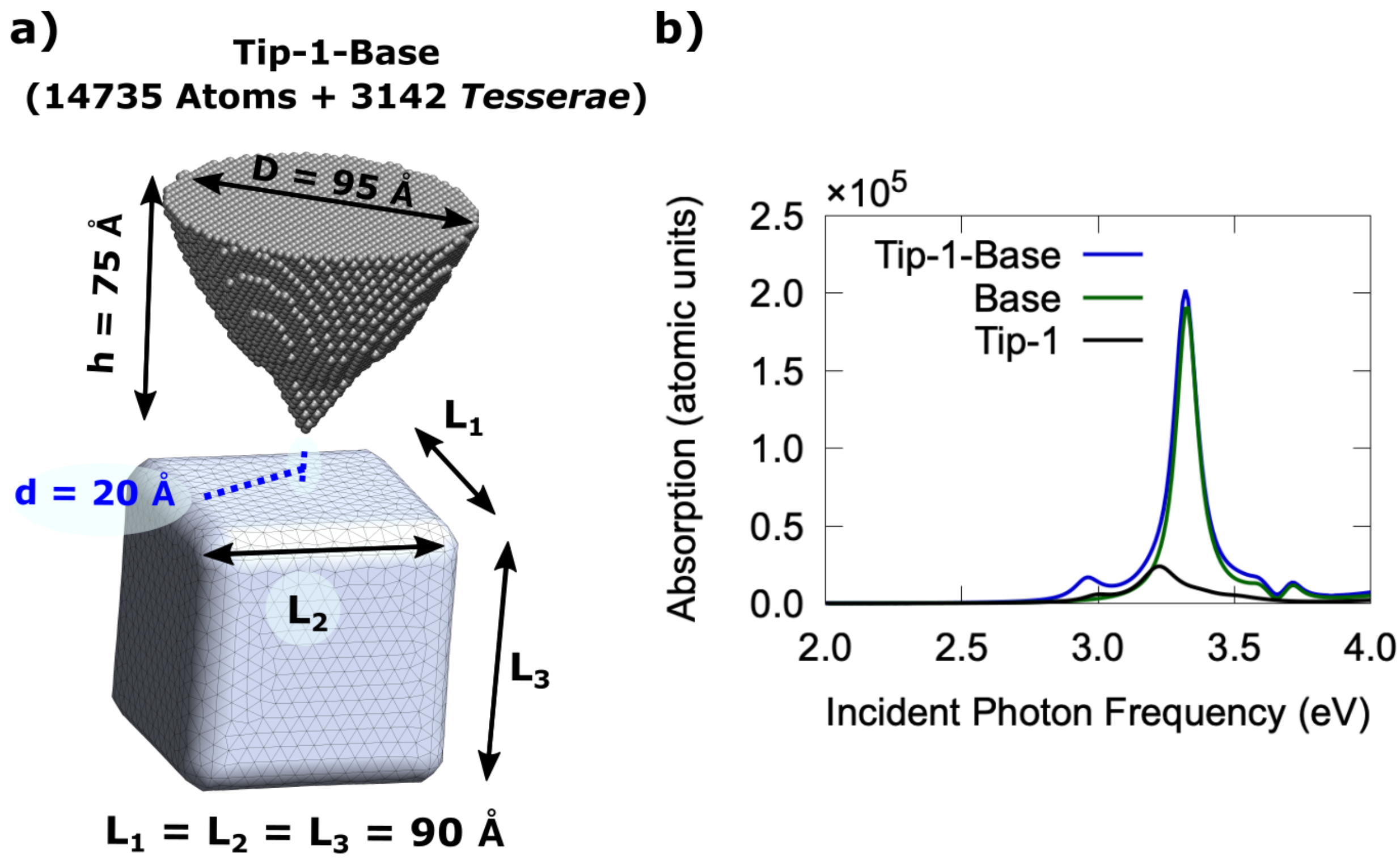

Figure S13: a) Silver ωFQFμ-BEM Tip1-base geometry. The ωFQFμ tip is positioned 20 Å above the base surface (BEM); the distance is calculated between the apex Ag atom and the closest base *tessera*. b) ωFQFμ-BEM absorption spectra (Tip1-base) compared with the ωFQFμ Tip-1 and the isolated BEM cube base ones.

To assess the effect of the base on the EET-mediated fluorescence intensity of the donor and acceptor molecules within the Tip1-base ωFQFμ-BEM calculations, we report in Fig. S14 a comparison of the simulated fluorescence spectra as a function of the tip position on top of the donor molecule for the ωFQFμ Tip-1 and ωFQFμ-BEM Tip1-base setups. We remark that the simulation frequency is $\omega = 2.5$ eV for

the Tip-1 simulations, while it is $\omega = 2.05$ eV for the Tip1-base simulations. This originates from the fact that the presence of the base further increases the coupling between the molecules and the metallic nanostructure, requiring a more off-resonant, red-shifted response, to achieve a better match with the experimental fluorescence pattern.

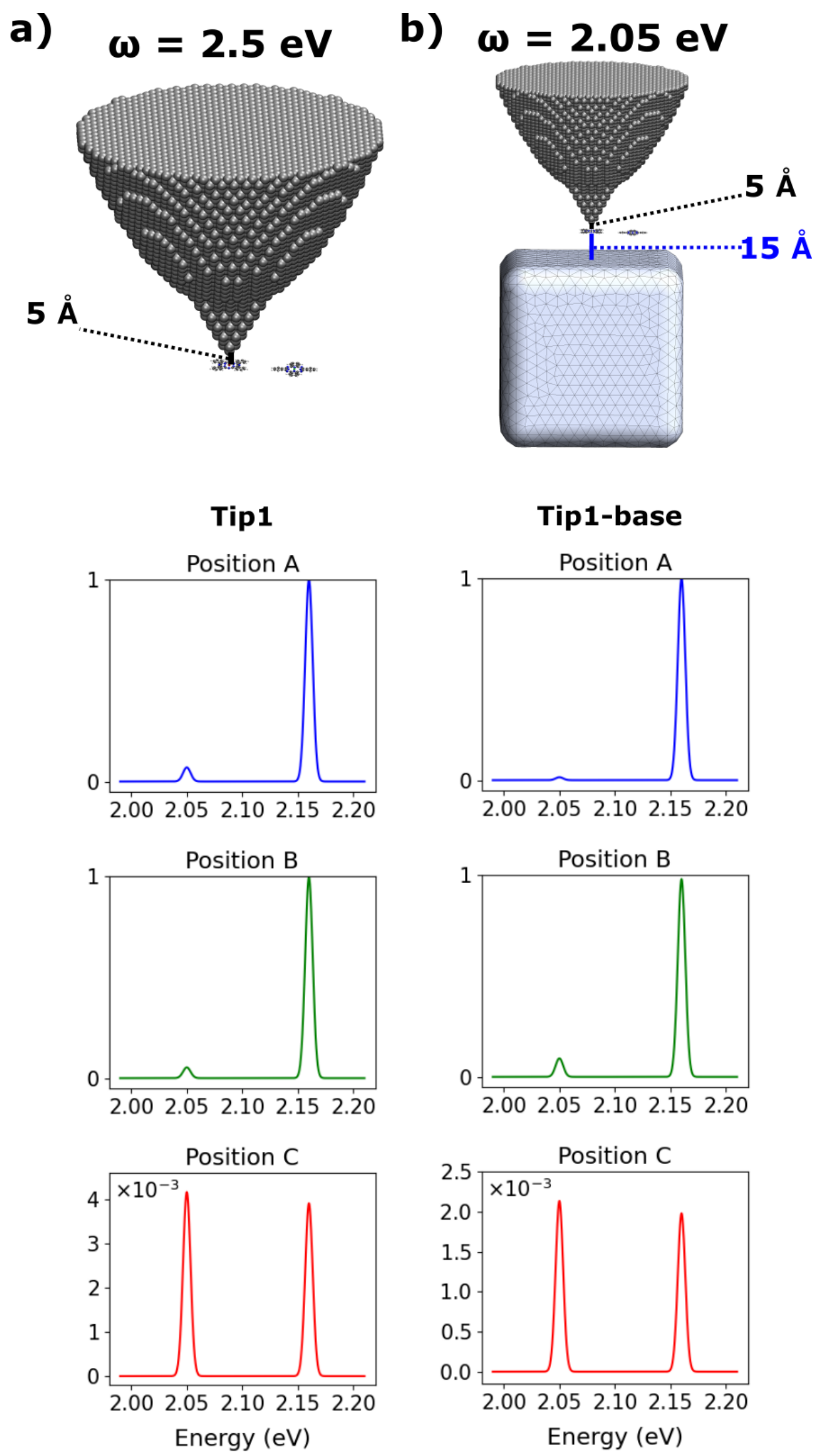

Figure S14: Geometries and normalized fluorescence spectra as a function of the tip position over the donor computed with TDDFT/ωFQFμ for Tip-1 (a) and Tip1-base (b).

In Fig. S15, we finally evaluate the effect of the tip–base distance on the Tip1-base absorption spectrum and on the EET-mediated fluorescence response with the tip at position C. As expected, the absorption

spectrum slightly increases in intensity and redshifts for the $d = 15$ Å setup relative to the $d = 20$ Å setup. Furthermore, considering the same simulation frequency, $\omega = 2.05$ eV, the Tip1-base setup at the shorter distance shows a clear mismatch with the experimental fluorescence pattern. This is due to the larger interaction between the base and the molecules, suggesting that a shorter tip–base distance would require a further off-resonant (red-shifted) simulation frequency to better match the experiment.

These results suggest that the base primarily introduces an overall shift in the coupling strength between the metallic nanostructure and the molecular donor–acceptor pair, rather than a qualitative change in the EET process itself; on this basis, we consider its effect on the atomistic-tip-morphology comparisons addressed in the main text to be negligible.

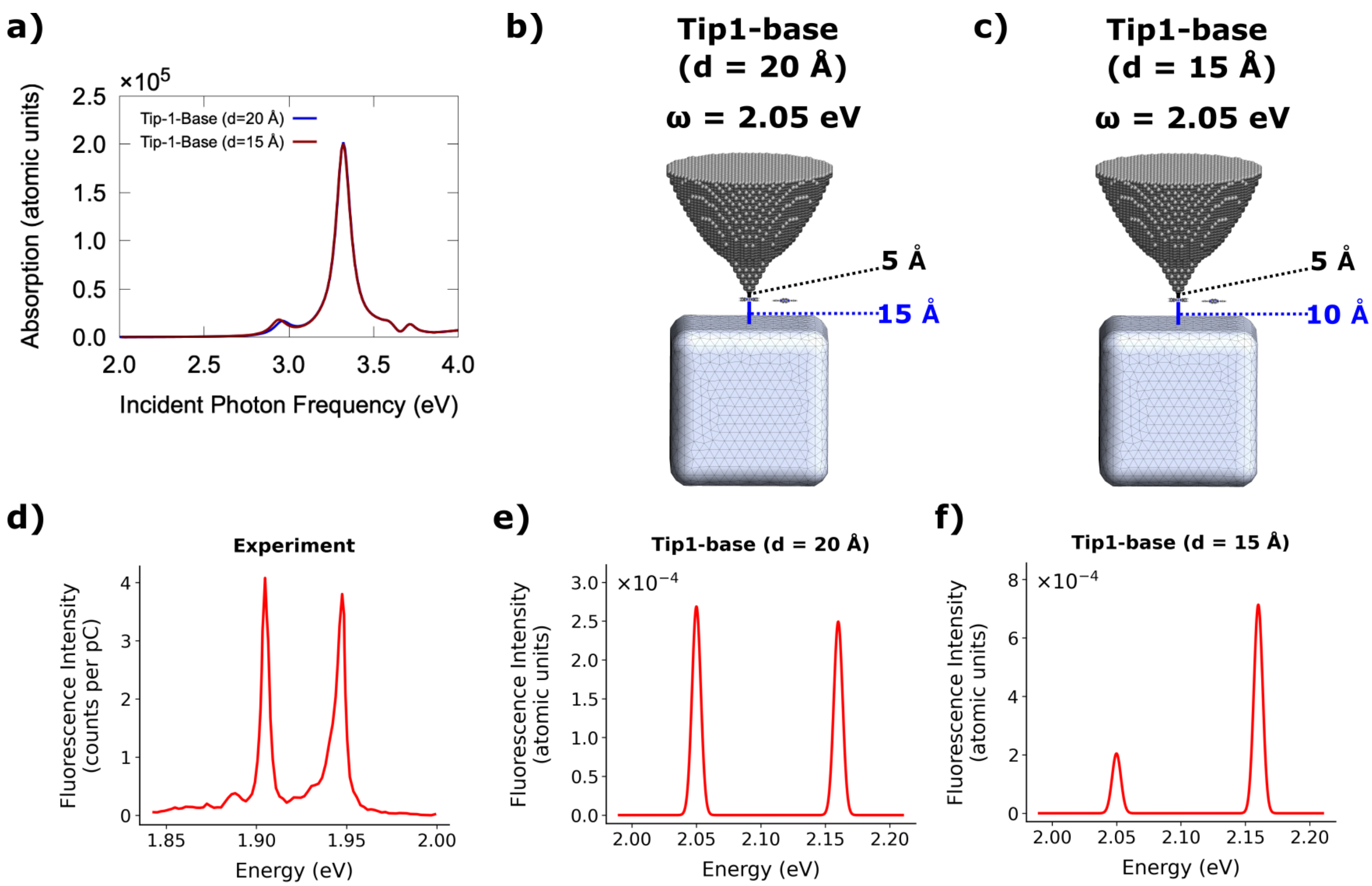

Figure S15: a) ωFQFμ-BEM absorption spectra of Tip1-base as a function of the tip-apex–to–base distance ($d = 20$ Å; $d = 15$ Å). b), c) Depiction of the tip-molecule-base arrangements used in the TDDFT/ωFQFμ-BEM simulations, with a tip-to-base total distance of $d = 20$ Å (b) and $d = 15$ Å (c). d) Experimental fluorescence spectrum reproduced with permission from Ref. 9 (Copyright 2022 Springer Nature) with the tip at position C, compared with the TDDFT/ωFQFμ-BEM fluorescence spectra of the Tip1-base geometry at $d = 20$ Å (e) and $d = 15$ Å (f).

References

(1) Giovannini, T.; Grobas Illobre, P.; Lafiosca, P.; Nicoli, L.; Bonatti, L.; Corni, S.; Cappelli, C. plasmonX: An open-source code for nanoplasmonics. *Computer Physics Communications* **2026**, *322*, 110035.

(2) Grobas Illobre, P.; Lafiosca, P.; Bonatti, L.; Giovannini, T.; Cappelli, C. Mixed atomistic–implicit quantum/classical approach to molecular nanoplasmonics. *J. Chem. Phys.* **2025**, *162*, 044103.

(3) Baerends, E.; et. al ADF *(version 2020.202)*. 2020; Theoretical Chemistry, Vrije Universiteit, Amsterdam, The Netherlands, http://www.scm.com, (accessed 2026-10-13).

(4) Grobas Illobre, P.; Lafiosca, P.; Guidone, T.; Mazza, F.; Giovannini, T.; Cappelli, C. Multiscale modeling of surface enhanced fluorescence. *Nanoscale Adv.* **2024**,

(5) Grobas Illobre, P. Electronic Energy Transfer Calculator. `https://github.com/pgrobasillobre/FretLab/releases/tag/v1.0.0`, 2026; GitHub Repository, (accessed 2026-10-13).

(6) Coane, C. V.; Romanelli, M.; Dall'Osto, G.; Di Felice, R.; Corni, S. Unraveling the mechanism of tip-enhanced molecular energy transfer. *Commun. Chem.* **2024**, *7*, 32.

(7) Grobas Illobre, P. GEOM: Nanostructure Geometry Generator. `https://github.com/pgrobasillobre/geom`, 2026; GitHub Repository, (accessed 2026-10-13).

(8) Geuzaine, C.; Remacle, J.-F. Gmsh: A 3-D finite element mesh generator with built-in pre- and post-processing facilities. *Int. J. Numer. Methods Eng.* **2009**, *79*, 1309–1331.

(9) Kong, F.-F.; Tian, X.-J.; Zhang, Y.; Zhang, Y.; Chen, G.; Yu, Y.-J.; Jing, S.-H.; Gao, H.-Y.; Luo, Y.; Yang, J.-L.; Dong, Z.-C.; Hou, J. G. Wavelike electronic energy transfer in donor–acceptor molecular systems through quantum coherence. *Nat. Nanotechnol.* **2022**, *17*, 729–736.

(10) Giovannini, T.; Bonatti, L.; Lafiosca, P.; Nicoli, L.; Castagnola, M.; Illobre, P. G.; Corni, S.; Cappelli, C. Do We Really Need Quantum Mechanics to Describe Plasmonic Properties of Metal Nanostructures? *ACS Photonics* **2022**, *9*, 3025–3034.

(11) Etchegoin, P. G.; Le Ru, E.; Meyer, M. An analytic model for the optical properties of gold. *J. Chem. Phys.* **2006**, *125*, 164705.

(12) Blaber, M. G.; Henry, A.-I.; Bingham, J. M.; Schatz, G. C.; Van Duyne, R. P. LSPR Imaging of Silver Triangular Nanoprisms: Correlating Scattering with Structure Using Electrodynamics for Plasmon Lifetime Analysis. *J. Phys. Chem. C* **2012**, *116*, 393–403.

(13) Johnson, P. B.; Christy, R.-W. Optical constants of the noble metals. *Phys. Rev. B* **1972**, *6*, 4370.

(14) Rakić, A. D.; Djurišić, A. B.; Elazar, J. M.; Majewski, M. L. Optical properties of metallic films for vertical-cavity optoelectronic devices. *Appl. Opt.* **1998**, *37*, 5271–5283.

(15) Johnson, P. B.; Christy, R.-W. Optical constants of the noble metals. *Phys. Rev. B* **1972**, *6*, 4370.

(16) Palik, E. D. *Handbook of optical constants of solids*; Elsevier, 1997.

(17) Vincett, P. S.; Voigt, E. M.; Rieckhoff, K. E. Phosphorescence and Fluorescence of Phthalocyanines. *The Journal of Chemical Physics* **1971**, *55*, 4131–4140.

(18) Romanelli, M.; Dall'Osto, G.; Corni, S. Role of metal-nanostructure features on tip-enhanced photoluminescence of single molecules. *J. Chem. Phys.* **2021**, *155*, 214304.

(19) Giovannini, T.; Rosa, M.; Corni, S.; Cappelli, C. A classical picture of subnanometer junctions: an atomistic Drude approach to nanoplasmonics. *Nanoscale* **2019**, *11*, 6004–6015.

(20) Giovannini, T.; Bonatti, L.; Polini, M.; Cappelli, C. Graphene plasmonics: Fully atomistic approach for realistic structures. *J. Phys. Chem. Lett.* **2020**, *11*, 7595–7602.

(21) Bonatti, L.; Gil, G.; Giovannini, T.; Corni, S.; Cappelli, C. Plasmonic resonances of metal nanoparticles: atomistic vs. Continuum approaches. *Front. Chem.* **2020**, *8*, 340.

(22) Lafiosca, P.; Giovannini, T.; Benzi, M.; Cappelli, C. Going Beyond the Limits of Classical Atomistic Modeling of Plasmonic Nanostructures. *J. Phys. Chem. C* **2021**, *125*, 23848–23863.

(23) Bonatti, L.; Nicoli, L.; Giovannini, T.; Cappelli, C. In silico design of graphene plasmonic hot-spots. *Nanoscale Adv.* **2022**, *4*, 2294–2302.

(24) Zanotto, S.; Bonatti, L.; Pantano, M. F.; Mišeikis, V.; Speranza, G.; Giovannini, T.; Coletti, C.; Cappelli, C.; Tredicucci, A.; Toncelli, A. Strain-Induced Plasmon Confinement in Polycrystalline Graphene. *ACS Photonics* **2023**, *10*, 394–400.

(25) Nicoli, L.; Lafiosca, P.; Grobas Illobre, P.; Bonatti, L.; Giovannini, T.; Cappelli, C. Fully atomistic modeling of plasmonic bimetallic nanoparticles: nanoalloys and core-shell systems. *Front. Photon.* **2023**, *4*, 2673–6853.

(26) Lafiosca, P.; Nicoli, L.; Pipolo, S.; Corni, S.; Giovannini, T.; Cappelli, C. Real-Time Formulation of Atomistic Electromagnetic Models for Plasmonics. *J. Phys. Chem. C* **2024**, *128*, 17513–17525.

(27) Giovannini, T.; Nicoli, L.; Corni, S.; Cappelli, C. The Electric Field Morphology of Plasmonic Picocavities. *Nano Lett.* **2025**, *25*, 10802–10808.

(28) Corni, S.; Tomasi, J. Enhanced response properties of a chromophore physisorbed on a metal particle. *J. Chem. Phys.* **2001**, *114*, 3739–3751.

(29) Tomasi, J.; Mennucci, B.; Cammi, R. Quantum Mechanical Continuum Solvation Models. *Chem. Rev.* **2005**, *105*, 2999–3094.

(30) Corni, S.; Tomasi, J. Excitation energies of a molecule close to a metal surface. *J. Chem. Phys.* **2002**, *117*, 7266–7278.

(31) Andreussi, O.; Corni, S.; Mennucci, B.; Tomasi, J. Radiative and nonradiative decay rates of a molecule close to a metal particle of complex shape. *J. Chem. Phys.* **2004**, *121*, 10190–10202.

(32) Lafiosca, P.; Nicoli, L.; Bonatti, L.; Giovannini, T.; Corni, S.; Cappelli, C. QM/Classical Modeling of Surface Enhanced Raman Scattering Based on Atomistic Electromagnetic Models. *J. Chem. Theory Comput.* **2023**, *19*, 3616–3633.

(33) Vukovic, S.; Corni, S.; Mennucci, B. Fluorescence enhancement of chromophores close to metal nanoparticles. Optimal setup revealed by the polarizable continuum model. *J. Phys. Chem. C* **2009**, *113*, 121–133.

(34) Sanchez-Gonzalez, A.; Corni, S.; Mennucci, B. Surface-Enhanced Fluorescence within a Metal Nanoparticle Array: The Role of Solvent and Plasmon Couplings. *J. Phys. Chem. C* **2011**, *115*, 5450–5460.

(35) Cao, S.; Rosławska, A.; Doppagne, B.; Romeo, M.; Féron, M.; Chérioux, F.; Bulou, H.; Scheurer, F.; Schull, G. Energy funnelling within multichromophore architectures monitored with subnanometre resolution. *Nat. Chem.* **2021**, *13*, 766–770.

(36) Angioni, A.; Corni, S.; Mennucci, B. Can we control the electronic energy transfer in molecular dyads through metal nanoparticles? A QM/continuum investigation. *Phys. Chem. Chem. Phys.* **2013**, *15*, 3294–3303.

(37) Doppagne, B.; Neuman, T.; Soria-Martinez, R.; López, L. E. P.; Bulou, H.; Romeo, M.; Berciaud, S.; Scheurer, F.; Aizpurua, J.; Schull, G. Single-molecule tautomerization tracking through space- and time-resolved fluorescence spectroscopy. *Nature Nanotechnology* **2020**, *15*, 207–211.

(38) Litman, Y.; Bonafe, F. P.; Akkoush, A.; Appel, H.; Rossi, M. First-Principles Simulations of Tip Enhanced Raman Scattering Reveal Active Role of Substrate on High-Resolution Images. *The Journal of Physical Chemistry Letters* **2023**, *14*, 6850–6859.

(39) Cui, J.; Zhang, Y.; Dong, Z.-C. Atomistic polarization model for Raman scattering simulations of large metal tips with atomic-scale protrusions at the tip apex. *Nanophotonics* **2023**, *12*, 4031–4042.
